# Heterogeneous integration of single-crystalline rutile nanomembranes with steep phase transition on silicon substrates

**DOI:** 10.1038/s41467-021-24740-2

**Published:** 2021-08-18

**Authors:** Dong Kyu Lee, Yunkyu Park, Hyeji Sim, Jinheon Park, Younghak Kim, Gi-Yeop Kim, Chang-Beom Eom, Si-Young Choi, Junwoo Son

**Affiliations:** 1 Department of Materials Science and Engineering, Pohang University of Science and Technology (POSTECH), Pohang, Republic of Korea; 2 Pohang Accelerator Laboratory, Pohang, Republic of Korea; 3 Department of Materials Science and Engineering, University of Wisconsin-Madison, Madison, WI USA

**Keywords:** Electronic properties and materials, Surfaces, interfaces and thin films, Electronic devices

## Abstract

Unrestricted integration of single-crystal oxide films on arbitrary substrates has been of great interest to exploit emerging phenomena from transition metal oxides for practical applications. Here, we demonstrate the release and transfer of a freestanding single-crystalline rutile oxide nanomembranes to serve as an epitaxial template for heterogeneous integration of correlated oxides on dissimilar substrates. By selective oxidation and dissolution of sacrificial VO_2_ buffer layers from TiO_2_/VO_2_/TiO_2_ by H_2_O_2_, millimeter-size TiO_2_ single-crystalline layers are integrated on silicon without any deterioration. After subsequent VO_2_ epitaxial growth on the transferred TiO_2_ nanomembranes, we create artificial single-crystalline oxide/Si heterostructures with excellent sharpness of metal-insulator transition ($\triangle \rho /\rho$ > 10^3^) even in ultrathin (<10 nm) VO_2_ films that are not achievable via direct growth on Si. This discovery offers a synthetic strategy to release the new single-crystalline oxide nanomembranes and an integration scheme to exploit emergent functionality from epitaxial oxide heterostructures in mature silicon devices.

## Abstract (web summary)

Unrestricted integration of single-crystal oxide films on Si substrates allows for exploitation of emerging functionality of new materials in mature silicon devices. Here the authors integrate epitaxial oxide films with sharp metal-insulator transition on Si substrates by epitaxial lift-off of a freestanding nanomembrane.

## Introduction

Heteroepitaxial growth has been widely used to obtain single-crystal films for developing modern solid-state electronic and photonic devices^1,2^. In particular, epitaxial oxide heterostructures provide promise for emerging electronics and photonics due to the intriguing phenomena (e.g., metal-insulator transition (MIT)^3–6^, high-electron mobility^7^, ferroelectricity^8^) exhibited by these ionic crystals. Therefore, the stacking of single-crystalline oxide films on dissimilar substrates (e.g., silicon (Si)) will offer ways to integrate the emergent phenomena of oxides with mature electronic and photonic devices^9–13^. However, the heteroepitaxial growth of oxide films drastically limits the possible material combinations due to the requirement of lattice matching between the epilayer and substrate^14^. For instance, direct oxide growth on dissimilar materials typically forms defective or polycrystalline layers near the interface between films and substrates^15,16^, preventing unrestricted integration of single-crystal oxide films onto any desired substrates, especially on mainstream Si substrates.

Release and transfer of freestanding single-crystal sheets with a nanoscale thickness (i.e., epitaxial lift-off for nanomembrane (NM)) gives the freedom to transfer the released epilayer onto highly mismatched or amorphous substrates, and even allows reusable substrates^9,17–21^. While crystalline materials that are intrinsically layered (e.g. two-dimensional (2D) materials) are exfoliated spontaneously due to weak van der Waals bonding between layers^22^, the freestanding NM from three-dimensional (3D) oxide crystals with strong bonding is hindered by the technical challenges of lifting strongly bonded epitaxial films from the oxide substrates. Thus, the technique for releasing the freestanding NM from a host substrate essentially requires the preferential creation of bond breaking from the substrate.

Many techniques have been employed to form freestanding NMs with a mechanically cleavable plane. For example, physical release methods (e.g., laser lift-off^20^) were originally developed to release epitaxial GaN semiconductor films to break strong bonding. However, these methods are only applicable to the formation of thick semiconductor membranes due to the inevitable structural damage. Moreover, a few monolayers of graphene could be inserted to release single-crystalline oxide membranes from substrates (i.e., “remote” epitaxy). Despite the versatility of this technique, the coalescence of localized nuclei in oxide layers on the graphene, along with the restriction of oxygen environment during growth, prevents the layer-by-layer growth of ultrathin oxide NM with atomic precision and high quality^10^.

By contrast, the freestanding NM was chemically released from the substrate by selective etching of sacrificial layers^18,19,21^; these chemical lift-off methods are less destructive than physical methods. However, the harsh wet condition with a strong acid or base etchant typically leaves roughening and residue on the host substrates or released membrane after the chemical etch^23,24^. Recently, atomically thin perovskite oxide NM was gently released by dissolving water-soluble Sr_3_Al_2_O_6_ sacrificial layers^11^, but the moisture-sensitive nature of these layers prevents long-time exposure of the sacrificial layers, which restricts practical application for heterogeneous integration of oxide NM. Moreover, the development of oxide NM has been limited for perovskite structure among chemical lift-off methods so far^10–12,21,25,26^; to extend the materials spectrum for freestanding oxide NM, a new combination of the sacrificial layer and etchant needs to be developed for the heterogeneous integration of epitaxial oxide NM with other crystal structures on dissimilar substrates^10^.

**Fig. 1:**
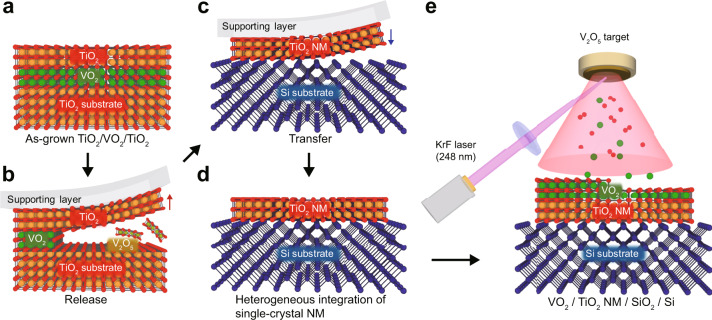
Process schematic for single-crystal rutile oxide nanomembranes (NM) on silicon. **a** Schematic of an epitaxial TiO_2_/VO_2_ heterostructure on TiO_2_ host substrate. **b** The VO_2_ layer is dissolved in H_2_O_2_ to release the top TiO_2_ film with the mechanical supporting layer. **c** The freestanding TiO_2_ NM is transferred onto the desired substrates (e.g., silicon). **d** By removing the rigid supporting layer, single-crystalline rutile oxide NM is heterogeneously integrated into a silicon substrate. **e** Epitaxial VO_2_ film with steep phase transition is grown on the TiO_2_-NM-templated Si substrates.

Here, we demonstrate that single-crystalline rutile oxide NM with the sharpened MIT can be integrated on the technologically influential, but more challenging, Si substrates by new epitaxial lift-off combination (see Fig. 1 for the process schematic). After the synthesis of an epitaxial TiO_2_/VO_2_ heterostructure on TiO_2_ host substrate (Fig. 1a), the VO_2_ sacrificial layer is selectively dissolved in dilute H_2_O_2_ to release the top TiO_2_ film with the mechanical supporting layers (Fig. 1b). Contrary to the previous chemical lift-off using extreme pH solution, dilute aqueous H_2_O_2_ with mild pH leads to selective etching of epitaxially grown VO_2_ sacrificial films by phase transformation to two-dimensional layered structure with weak bonding; this selective dissolution of VO_2_ layer releases millimeter-scale freestanding TiO_2_ NM from the TiO_2_/VO_2_/TiO_2_ heterostructures at room temperature. Then, the single-crystalline TiO_2_ NM is transferred onto the Si substrates without any deterioration of crystal quality (Fig. 1c, d). Interestingly, transferred TiO_2_ single-crystal NM serves as a template for the heterogeneous integration of single-crystal VO_2_ films on Si substrate (Fig. 1e). As a result of the VO_2_ film epitaxially grown on TiO_2_ NM template, more than three orders of magnitude modulation of resistivity ratio ($\triangle \rho /\rho$ > 10^3^) and sharpened MIT (i.e., narrow FWHM of Gaussian fitting from d(log_10_($\rho$))/d*T* during heating and cooling ($\triangle {T}_{{{{\mathrm{h}}}}}$ and $\triangle {T}_{{{{\mathrm{c}}}}}$ ~3 K)) are remarkably achieved even in the ultrathin (≤10 nm) VO_2_ films on Si substrates, which is not possible using conventional thin film growth.

## Results

### Synthesis of freestanding single-crystalline TiO_2_ nanomembranes

**Fig. 2:**
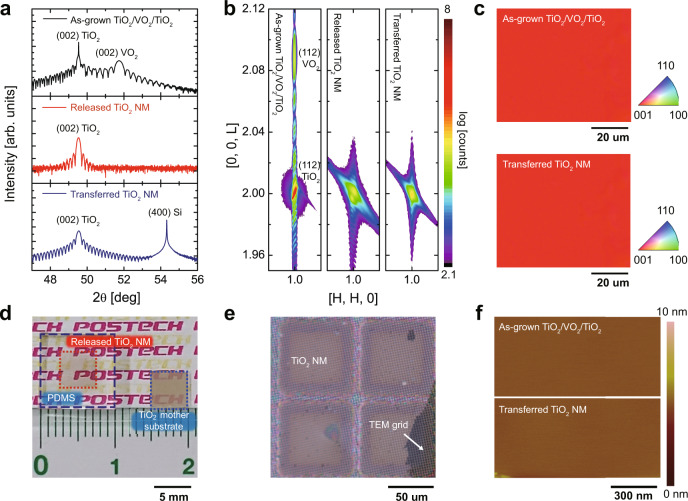
Crystal and surface quality of released and transferred TiO_2_ single-crystal nanomembrane (NM). **a** Symmetric 2$\theta -\omega$ scan. **b** Reciprocal space mapping around (112) reflection of as-grown TiO_2_ epitaxial films and TiO_2_ single-crystal NM released on supporting layer and transferred on Si substrates. The high-resolution X-ray diffraction measurements show single-crystallinity with uniform out-of-plane orientation and no in-plane rotation in released and transferred TiO_2_ NM. **c** EBSD maps of the as-grown TiO_2_ epitaxial films on VO_2_/TiO_2_ substrates (top) and transferred TiO_2_ NM on Si substrates (bottom), confirming the single-crystalline out-of-plane orientation. **d** Photograph of 40-nm-thick TiO_2_ single-crystal NM released on mechanical support with the identical lateral dimension with TiO_2_ host substrates. **e** OM image of transferred TiO_2_ NM on the carbon TEM grid. **f** AFM image of as-grown TiO_2_ films and transferred TiO_2_ NM, which confirms a uniform and flat surface without surface cracks or residues.

Prior to release and transfer of TiO_2_ NM, TiO_2_ (10–70 nm)/VO_2_ (~17 nm) heterostructures were epitaxially grown on (001)-oriented TiO_2_ substrates by pulsed laser deposition (PLD) (Fig. 1a). Symmetrical 2θ-ω scan using synchrotron x-ray scattering on TiO_2_/VO_2_/TiO_2_ detected two (002)_R_ Bragg reflections, one from the rutile TiO_2_ (2θ = 49.54°) film and substrate, and one from VO_2_ film (2θ = 51.75°) (black line in Fig. 2a)^27^. Due to the close resemblance in crystal structure and small in-plane lattice mismatch of (001)_R_-VO_2_ and (001)-TiO_2_ (${f}_{a}\approx$ 0.86% along a- and b-axis), 17-nm-thick (001)_R_-VO_2_ films are fully strained by biaxial tensile strain; all heterostructure maintained identical in-plane lattice constant (left figure of Fig. 2b). Thus, the thickness of VO_2_ (~17 nm) was determined to create unstrained and defect-free TiO_2_ epitaxial films regardless of film thickness^27,28^.

Then, rigid support layer (e.g., PDMS) was coated on the heterostructure before release to mechanically stabilize the TiO_2_ NM and to facilitate the subsequent transfer of the released NM^10–12,25^. By simply immersing these TiO_2_/VO_2_/TiO_2_ heterostructures into the dilute (10%) hydrogen peroxide (H_2_O_2_) solution at room temperature (Fig. 1b), a few millimeter-scale TiO_2_ films were successfully released from their substrates by fully dissolving VO_2_ sacrificial layers (i.e., denoted as released TiO_2_ NM), allowing the growth substrate to be removed. The released single-crystal NM was transferred on any arbitrary substrate (i.e., denoted as transferred TiO_2_ NM), including on SiO_2_-coated (~80 nm) Si (100) substrates (Fig. 1c). The transferred NM was then heated at 60 °C to form stronger adhesion with substrates and the support layer was slowly detached (Fig. 1d).

Interestingly, the VO_2_ sacrificial layer was dissolved selectively and rapidly in the dilute H_2_O_2_ solution with mild pH (~5.3) (Fig. 1b). The combination of the VO_2_ sacrificial layer and H_2_O_2_ etchant for single-crystal rutile oxide NM in terms of release rate is as effective as that of a Sr_3_Al_2_O_6_ sacrificial layer and H_2_O for a single-crystal perovskite oxide NM^11^. The high etching capability of H_2_O_2_ on VO_2_ was confirmed by direct comparison with that of an HNO_3_ solution with the same concentration as a well-established VO_2_ etchant with a strong acid (pH ~2)^24^ (Supplementary Fig. 1): While the residue of VO_2_ films remained even after 64 s after VO_2_ films were immersed into strong acid HNO_3_ solution, the H_2_O_2_ solution completely removed VO_2_ films that were free of any residue on the surface of the TiO_2_ substrate within 4 s. Furthermore, repeating H_2_O_2_ etching preserves the atomically flat surface topography, implying substrate reusability for the production of TiO_2_ NM (Supplementary Fig. 2). VO_2_ films could be spontaneously oxidized by H_2_O_2_ and transformed to V_2_O_5_ and/or water-soluble ${{V}_{2}O}_{5}\cdot{nH}_{2}O(s)$ gels with a layered van der Waals (vdW) structure along the c-axis^29,30^; these layered crystals with weak bonding are exfoliated and dispersed in the solution (Supplementary Fig. 3a, b). Since unreacted TiO_2_ epitaxial layers cover whole surfaces of VO_2_ sacrificial layers during the release process, the release of epitaxial TiO_2_ NM by oxidation and dissolution of VO_2_ sacrificial layers begins from the edge of the substrate (i.e., side etching); the dissolution (or release) time increases with the lateral size of the TiO_2_ substrate (Supplementary Fig. 4).

As a result of effective dissolution of the VO_2_ sacrificial layer by H_2_O_2_, the quality and alignment of epitaxial TiO_2_ layers are intact during the release and transfer process as observed in symmetric 2θ-ω scans using synchrotron X-ray scattering (Fig. 2a). While the (002)_R_ peak from the VO_2_ sacrificial layer (2θ = 51.75°) was clearly removed after the selective etching process (red line in Fig. 2a), the (002)_R_ peak from ~ 40-nm-thick (001) TiO_2_ single-crystal films with thickness oscillations was observed consistently at 2θ = 49.54° in released (red line in Fig. 2a) and transferred TiO_2_ NM on the Si substrate (blue line in Fig. 2a and Supplementary Fig. 5 with Cu K_α1_ X-ray radiation). Moreover, the (002)_R_ peak from TiO_2_ NM simultaneously appeared with a (400) peak from the Si substrate (2θ = 54.32°) in the transferred TiO_2_ NM (blue line in Fig. 2a), which represents that the c-axis of single-crystal TiO_2_ NM was aligned with the out-of-plane orientation of the Si substrate. Azimuthal X-ray diffraction (XRD) $\phi$ scanning of (112)_R_ crystallographic plane showed identical fourfold symmetry of released and transferred TiO_2_ NMs with those of the single-crystal (001) TiO_2_ host substrate (Supplementary Fig. 6); this result showed in-plane single-crystallinity of NMs without any rotated domains. Additionally, an electron backscatter diffraction (EBSD) map confirmed (001) orientation of rutile crystals over a large area in both as-grown and transferred TiO_2_ layers (Fig. 2c).

To obtain more detailed information on the crystal structures of TiO_2_ NMs during the release and transfer process, both in-plane and out-of-plane lattice parameters were monitored using reciprocal space mapping (RSM) around the (112) reflection of as-grown, released, and transferred 70-nm-thick TiO_2_ layers (Fig. 2b). The RSM data clearly show sharp and intense (112) Bragg reflections and Kiessig fringes from the TiO_2_ layer and substrates, and from the VO_2_ sacrificial layers in as-grown heterostructures. Since 17-nm-thick VO_2_ sacrificial layers are coherently grown on the TiO_2_ substrates with identical H (i.e., in-plane reciprocal space unit), strain-free epitaxial TiO_2_ layers with various thickness (10–70 nm) are coherently grown on VO_2_/TiO_2_ substrates (Fig. 2b and Supplementary Fig. 7b, c). After selective etching of the VO_2_ sacrificial layer, H and L in released and transferred TiO_2_ NM was identical with those in TiO_2_ substrates and films in as-grown heterostructures; these strain-free TiO_2_ layers with precisely controlled thickness are released and transferred onto a Si substrate without modification of the lattice parameters and crystallinity (Supplementary Fig. 7a).

Furthermore, surfaces of the released and transferred TiO_2_ NM are uniform and intact without any defective boundaries^31^. Figure 2d shows that the entire area of TiO_2_ films with lateral dimension of millimeter-scale and nanometer thickness was fully released from the VO_2_/TiO_2_ substrates after this etching process. Optical microscope (OM) images of transferred TiO_2_ NM on the carbon TEM grid exhibit crack-free layers with natural wrinkles (Fig. 2e and Supplementary Fig. 8a). Both scanning electron microscope (SEM, Supplementary Fig. 8b) and atomic force microscope (AFM, Fig. 2f) images confirmed uniform and flat surface of both released and transferred TiO_2_ NM without surface cracks or residues: the arithmetic average surface roughness of transferred TiO_2_ NM (R_a_ = 0.06 nm) was comparable to that of originally grown TiO_2_ films on VO_2_/TiO_2_ substrates (R_a_ = 0.04 nm), representing residue-free VO_2_ removal by H_2_O_2_ etching and subsequent surface cleaning (Fig. 2f and Supplementary Figs. 9, 10).

**Fig. 3:**
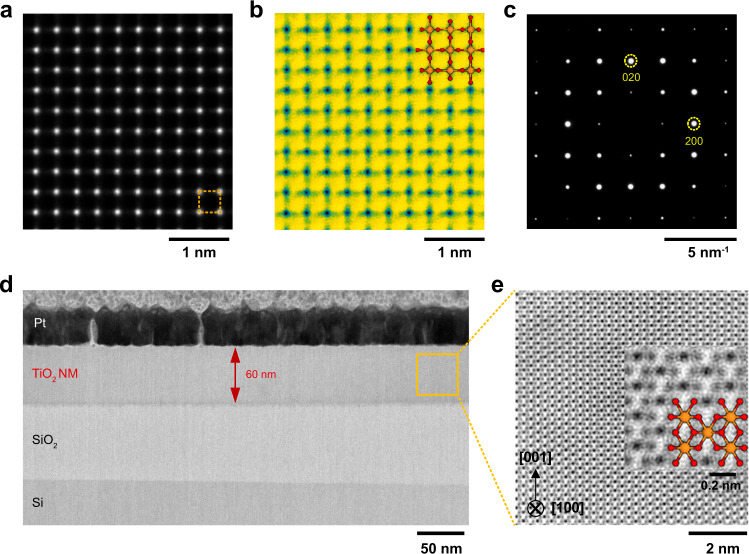
STEM analysis of freestanding single-crystal TiO_2_ nanomembrane (NM). **a** HAADF-STEM, **b** ABF-STEM, and **c** SADP images from the plane-view observation on 20-nm-thick freestanding TiO_2_ NM. The white spots in HAADF-STEM, which are identical to Ti atoms, are consistent with cation sites of [001]_R_-projected rutile structure. The atomic arrangement of ABF-STEM, visualization of oxygen atoms, was also identical to anion sites of rutile structure; the results indicate that atomic arrangement of freestanding TiO_2_ NM was identical to the single-crystal rutile structure without defective features. **d**, **e** are the cross-sectional ABF-STEM images of TiO_2_ NM, the quality of which is crystallographically high with perfect registry of Ti (orange color in **e**) and O (red color in **e**) atoms in rutile structure.

High-resolution scanning transmission electron microscopy (STEM) confirms the local atomic structure of freestanding single-crystal TiO_2_ NM. First, the released TiO_2_ NM was transferred onto a TEM grid for STEM observation (Fig. 2e and Supplementary Fig. 11); the high-angle annular dark-field (HAADF) and the annular bright-field (ABF) STEM were applied for the plane-view observation. The HAADF-STEM image shows a square pattern of four titanium atoms (orange, dotted square) without a lattice distortion or defects (Fig. 3a). More interestingly, the ABF-STEM image shows the contrast tails from the titanium atom with contribution by the oxygen atoms and the pattern exactly matched the atomic structure of the rutile TiO_2_ with the [001] zone axis (Fig. 3b). The selected-area diffraction pattern (SADP) provided that the in-plane lattice parameters of TiO_2_ NM were 0.46 nm (Fig. 3c), which confirms the freestanding TiO_2_ film was perfectly transferred. After the TiO_2_ NM (~60 nm) was transferred to the SiO_2_/Si substrate, cross-sectional STEM observation was performed with the [100] zone axis to visualize the structural coherency of the freestanding oxide NM, as shown in Fig. 3d, e. The low magnification ABF-STEM image shows that the TiO_2_ NM is free of defects even after its transfer process from the wide field of view (Fig. 3d); the high magnification ABF-STEM image again verifies the perfect registry of Ti (orange) and oxygen (red) atoms in the rutile structure (Fig. 3e).

### Heterogeneous integration of single-crystalline VO_2_ films on the TiO_2_-NM-templated Si substrates

**Fig. 4:**
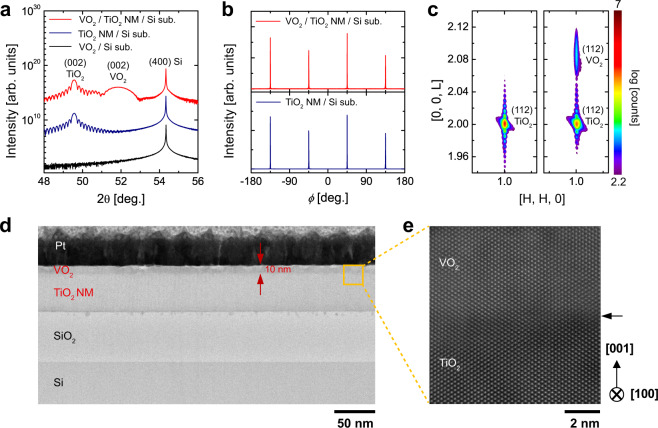
Heterogeneous integration of rutile oxide epitaxial VO_2_/TiO_2_ heterostructures on Si substrates. **a** Symmetric 2$\theta -\omega$ scan, **b** asymmetric $\phi$ scan, and **c** reciprocal space mapping around (112) reflection of TiO_2_ NM on Si substrates before (blue in **a**, **b**, and left in **c**) and after (red in **a**, **b**, and right in **c**) the growth of VO_2_ films, implying single-crystallinity of coherently strained VO_2_ films on TiO_2_ NM/Si. **d** The cross-sectional ABF-STEM images of the heterogeneous VO_2_/TiO_2_ NM on SiO_2_/Si. The atomic-scale HAADF-STEM image at the area indicated by the yellow square in **d** is shown in **e** (zone axis: [100] in TiO_2_).

After transfer to the (SiO_2_-coated) Si substrates, the single-crystalline TiO_2_ NMs served as templates for the epitaxial growth of high-quality VO_2_ thin films on Si substrates (Fig. 1e). As observed in symmetric 2θ – ω scans, the intense (002)_R_ VO_2_ peak appeared at ~2θ = 51.85° along with peaks related to the TiO_2_ template (~2θ = 49.54°) and Si substrates (~2θ = 54.34°) for the VO_2_ films on the TiO_2_-templated Si substrates (red line in Fig. 4a), compared to the absence of related peaks for the VO_2_ films directly grown on Si substrate (black line in Fig. 4a); this result reveals that lattice-matched single-crystal templates facilitate the formation of epitaxial VO_2_ films on Si substrates. Single-crystallinity of VO_2_ films on TiO_2_ NM/Si was again verified by identical fourfold symmetry with TiO_2_ NM templates in asymmetric $\phi$-scans of the (112)_R_ plane (Fig. 4b). While the out-of-plane strain state of the TiO_2_ NM did not change after the growth of VO_2_ films, the out-of-plane lattice parameters of VO_2_ films (2.838 Å) were reduced compared to those of bulk VO_2_ (2.88 Å). Indeed, an RSM near the (112) reflection of the TiO_2_ NM confirms that the peaks from the VO_2_ films and TiO_2_ NM showed identical H (i.e., in-plane reciprocal space unit) (Fig. 4c), which indicates that a 10-nm-thick VO_2_ film remains coherently strained to the TiO_2_ NM along the in-plane direction^27,28^.

Low magnification ABF- and high magnification HAADF-STEM images (Fig. 4d, e) confirm that the epitaxial growth of 10-nm-thick VO_2_ on TiO_2_ NM can realize heterogeneous integration of single-crystal VO_2_ films on a Si substrate. In particular, VO_2_ and TiO_2_ are coherently matched and the interface can be visualized due to the slight contrast difference between VO_2_ and TiO_2_, as indicated by the arrow (Fig. 4e); the atomic resolution image implicates that the VO_2_ layer is tightly constrained from the underlying TiO_2_ NM, and thus TiO_2_ NM templates between VO_2_ and Si enable the growth of epitaxial VO_2_ films with crystallographic perfection free from defects.

**Fig. 5:**
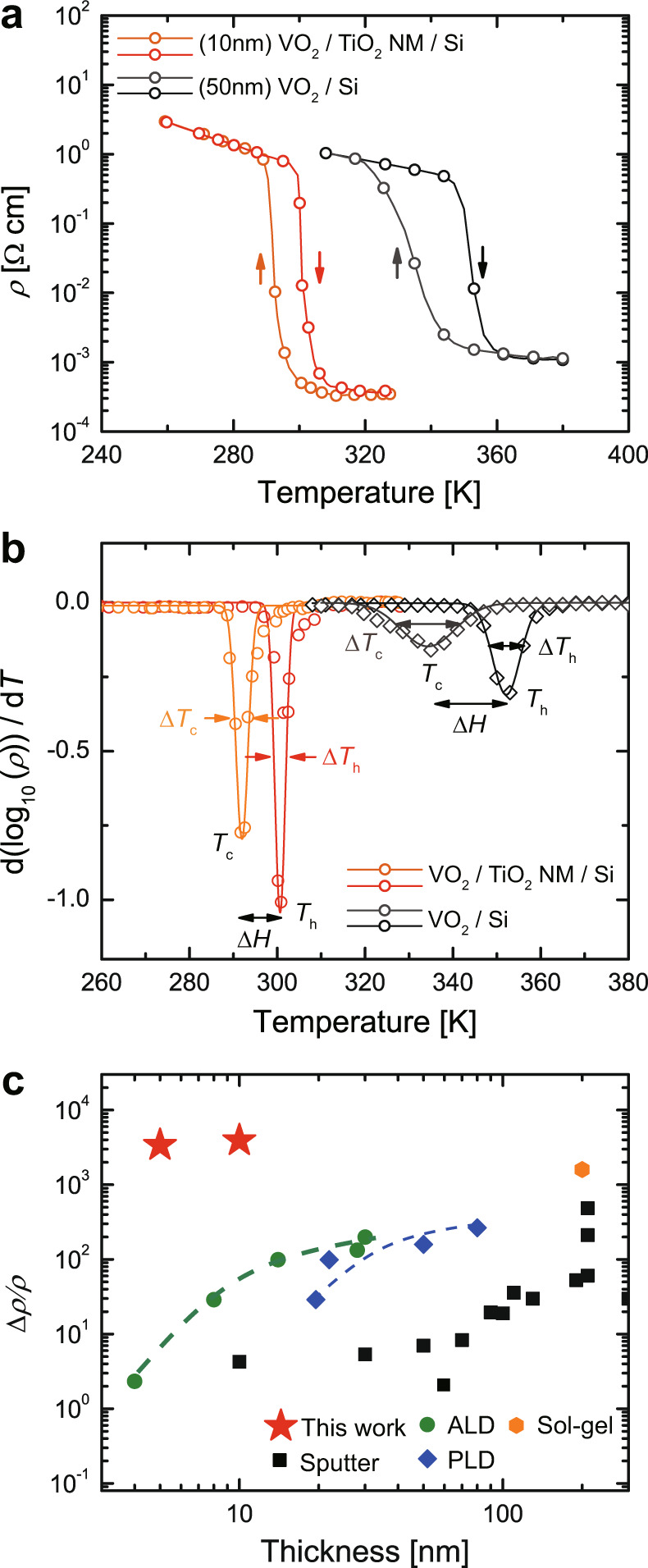
Single-crystalline VO_2_ films with steep phase transition on TiO_2_-NM-templated Si substrate. **a** Temperature-dependent resistivity modulation near *T*_MIT_ in 10-nm-thick VO_2_ films on TiO_2_ NM/SiO_2_/Si (red lines) and 50-nm-thick VO_2_ films on SiO_2_/Si (black lines). **b** Comparison of various metal-insulator transition properties (d(log_10_($\rho$))/d*T*, *T*_h_, *T*_c_, $\triangle H$, $\triangle {T}_{{{{\mathrm{h}}}}}$, $\triangle {T}_{{{{\mathrm{c}}}}}$) between 10-nm-thick VO_2_ films on TiO_2_ NM/SiO_2_/Si (red lines) and 50-nm-thick VO_2_ films on SiO_2_/Si (black lines). Note that the transition width and sharpness, as well as the resistivity ratio, were substantially improved in VO_2_ films with TiO_2_ NM, compared to those without TiO_2_ NM. **c** Benchmark of resistivity ratio $\Delta \rho /\rho =({\rho }_{{T}_{{{{{\mathrm{MIT}}}}}}-15K}-{\rho }_{{T}_{{{{{\mathrm{MIT}}}}}}+15K})/{\rho }_{{T}_{{{{{\mathrm{MIT}}}}}}+15K}$ for VO_2_ films on Si substrates across the MIT. For a direct comparison, all films were grown on oxide-coated Si substrates using various growth techniques: (sputtering (black square), ALD (green circle), PLD (blue diamond), and sol-gel (orange hexagon)) reported in the previous literature (Supplementary Table 1). While deteriorated $\triangle \rho /\rho$ was observed in VO_2_ films directly grown on Si due to the polycrystallinity of the films and the formation of a defective interfacial layer, epitaxial growth of VO_2_ films (5 and 10 nm) guided by transferred TiO_2_ NM enables integration of correlated oxides on silicon substrates with the highest modulation of resistivity ratio across the metal-insulator transition (red stars).

To benchmark the quality of the VO_2_ thin films on the TiO_2_-NM-templated SiO_2_/Si substrates in terms of MIT, temperature-dependent resistivity was measured for VO_2_ films depending on the existence of transferred TiO_2_ NM. Higher resistivity modulation ($\triangle \rho /\rho$ ~ 3.3 × 10^3^) was observed across *T*_MI_ ~ 296 K in both 5-nm-thick and 10-nm-thick VO_2_ on TiO_2_ NM/SiO_2_/Si (red line in Fig. 5a and Supplementary Fig. 13b) than in 10-nm-thick VO_2_ on SiO_2_/Si (Supplementary Fig. 13a) and even in 50-nm-thick VO_2_ on SiO_2_/Si ($\triangle \rho /\rho$ ~ 5.2 × 10^2^ across the *T*_MI_ ~ 343 K) (black line in Fig. 5a). In addition to the resistivity ratio, other MIT properties (d(log_10_($\rho$))/d*T*, *T*_h_, *T*_c_, $\triangle H$, $\triangle {T}_{{{{\mathrm{h}}}}}$, $\triangle {T}_{{{{\mathrm{c}}}}}$) were characterized as shown in Fig. 5b^32^. d(log_10_($\rho$))/d*T* was plotted and fitted with a Gaussian function. *T*_h_ and *T*_c_ were then defined at the peak position of the Gaussian and the transition width was calculated from the difference ($\triangle H$ = *T*_h_ − *T*_c_). The transition sharpness $\triangle {T}_{{{{\mathrm{h}}}}}$ and $\triangle {T}_{{{{\mathrm{c}}}}}$ was estimated from the full-width at half-maximum of the Gaussian peak. In fact, the transition width and sharpness, as well as the resistivity ratio, were significantly improved in the VO_2_ films with TiO_2_ NM (i.e., 8.70 and 3.3 K for $\triangle H$ and $\triangle {T}_{{{{\mathrm{c}}}}}$, respectively) compared to those without TiO_2_ NM (i.e., 17.94 and 14.54 K for $\triangle H$ and $\triangle {T}_{{{{\mathrm{c}}}}}$, respectively). The sharpened phase transition is attributed to the single-crystalline nature of the VO_2_ films perfectly aligned with the underlying single-crystal TiO_2_ NM templates. Moreover, the transferred single-crystal TiO_2_ NM forms coherently tensile-strained VO_2_ films; this epitaxial strain leads to a *T*_MI_ shift close to room temperature in VO_2_ films on TiO_2_ NM/SiO_2_/Si compared to relaxed VO_2_ films on SiO_2_/Si substrates^27,28,32^. Along with steep MIT under temperature, these single-crystalline VO_2_ films on TiO_2_ NM/Si show high endurance during thermal and electrical cycling. Both thermally induced MIT and electrically induced MIT (I_on_/I_off_ > 10^3^) were consistently observed without any drift during the multiple cycles of thermal switching (Supplementary Fig. 14) and electrical switching (Supplementary Fig. 15), respectively.

## Discussion

It should be noted that the integration of VO_2_ films with excellent electrical properties on a Si substrate has proved challenging due to fundamental limitations. A representative set of data of MIT properties from VO_2_ thin films on Si (or oxide-coated Si or buffered Si) substrates was compiled to allow for a direct comparison with those from our films (Fig. 5c, Supplementary Table 1, and Supplementary Fig. 16). Despite previous attempts to directly grow VO_2_ thin films on Si substrates using various deposition techniques, $\triangle \rho /\rho$ across the *T*_MI_ deteriorated due to the formation of polycrystallinity of the films. Notably, a further reduction in $\triangle \rho /\rho$ was found to occur with decreasing film thickness, likely due to the formation of defective interfacial layers (e.g., by thermodynamic reaction of VO_2_ with Si (or SiO_2_)); the cation-to-anion stoichiometry was not maintained in the VO_2_ films near the interface^33,34^. If this compositional variation (i.e., off-stoichiometry) contains a significant fraction of the films, the resistivity ratio and transition sharpness will significantly degrade in the regime of an ultrathin thickness (<40 nm), as observed in our 10-nm-thick VO_2_ films on SiO_2_/Si and Al_2_O_3_-buffered Si (Supplementary Figs. 13, 16); steeper transition cannot be engineered simply by direct deposition of thin VO_2_ films on Si substrates due to the existence of interfacial layers and the substantial density of defects.

However, the TiO_2_ template NM on Si substrates permits epitaxy with VO_2_ thin films due to its identical crystal structure (P4_2_/mnm) and small lattice mismatch (*f*_*a*_ ~ 0.86%). The transferred NM could play a crucial role as a seed layer for epitaxy, and artificially allows the first demonstration on heterogeneous integration of single-crystalline VO_2_ on Si substrates, which are commonly employed in electronic devices. Furthermore, the TiO_2_ NM is likely to prevent subsequent reaction between VO_2_ and the Si substrate, and allows excellent control over the V-oxidation states without any extended defects^33,34^. Thus, epitaxial growth of ultrathin VO_2_ films guided by transferred TiO_2_ NM enables integration of correlated oxides with unprecedented modulation of the resistivity ratio ($\triangle \rho /\rho$ > 10^3^) across the MIT in the regime of an ultrathin thickness (5–10 nm) on Si substrates (see red stars in Fig. 5c).

In summary, heterogeneous integration of a freestanding single-crystalline rutile oxide NM was achieved by exploiting selective oxidation and dissolution of isostructural sacrificial layers. Despite a mild pH condition under a dilute H_2_O_2_ solution at room temperature, VO_2_ sacrificial films are spontaneously oxidized by H_2_O_2_ and transformed to layered crystals, which in turn could be exfoliated and dispersed in solution to release and transfer the millimeter-scale TiO_2_ NM with controlled thickness on oxide-coated Si substrates. Owing to the nearly perfect single-crystallinity of the transferred rutile TiO_2_ NM, this lattice-matched single-crystal template permits heterogeneous integration of epitaxial VO_2_ films on Si substrates; exceptional MIT characteristics in terms of transition sharpness ($\triangle {T}_{h}$ ~ 2.85 K and $\triangle {T}_{c}$ ~ 3.3 K) and resistivity ratio ($\triangle \rho /\rho$ > 10^3^) were realized in ultrathin VO_2_ films integrated on Si substrates, benefitting from the superior quality of epitaxial oxide films.

Our strategy to release and transfer a freestanding epitaxial rutile oxide NM for a MIT will provide an unprecedented platform using emergent phenomena in epitaxial oxide heterostructures to be integrated with current state-of-the-art Si-based technology (e.g., integrated electronics^3,6^ and photonics^13,35^ on Si). For example, our epitaxial oxide NM with steep phase transition could deliver a hybrid optical modulator with a high extinction ratio, low loss, and high modulation speed integrated on Si waveguides^13,35,36^. Moreover, our approach for single-crystalline oxide NMs with high thermal stability are generally applicable for heterogeneous integration of high-quality oxide NM with any rutile crystal structures on Si substrates (e.g., epitaxial growth of metallic RuO_2_ single-crystalline NMs on transferred TiO_2_ NM/Si or direct transfer of metallic RuO_2_ single-crystal NMs on Si, Supplementary Figs. 17–20); our study enables us to extend the materials spectrum for freestanding single-crystalline rutile oxide NM by developing a new combination of sacrificial layer and etchant. It also offers a unique opportunity for new types of artificial heterostructures by stacking multi-functional oxide NMs with other 2D/3D single-crystalline NMs (e.g., III–V, exfoliated 2D layered materials, complex oxides)^10,17^ or by controlling twisted angle between two misaligned sheets of oxide NMs (similar to twisted bilayer graphene heterostructure with atomic and electronic reconstruction)^37,38^ for novel interfacial physics and a new generation of emergent devices.

## Methods

### Epitaxial film growth

The epitaxial VO_2_ thin films (~17 nm) were grown on (001) TiO_2_ single-crystal substrates, followed by the growth of TiO_2_ films (10–70 nm) by PLD. First, (001) TiO_2_ single-crystal substrates (Shinkosha CO., LTD) with lateral size of up to 5 mm × 5 mm were loaded into the PLD chamber, which was then evacuated to a base pressure of ~1 × 10^−6^ Torr. The rotating V_2_O_5_ and TiO_2_ targets were then ablated by focusing a KrF excimer laser (Coherent Complex Pro 102 F, $\lambda$ = 248 nm) with a fluence of 1 J/cm^2^ and a repetition rate of 1 Hz. The growth of VO_2_ films was performed at fixed ${P}_{{O}_{2}}$ = 15 mTorr and *T*_*g*_ = 300 °C, which were selected to induce a steep MIT near room temperature from coherently tensile-strained VO_2_ films. Subsequently, TiO_2_ films were grown on VO_2_ templates under ${P}_{{{{{\mathrm{O}}}}}_{2}}$ = 28 mTorr and *T*_*g*_ = 300 °C. After the growth of heterostructure, the samples were cooled to room temperature at a rate of 20 °C/min. The epitaxial VO_2_ film (5–10 nm) was grown on the transferred TiO_2_ NM/Si with an identical growth condition of that of VO_2_ sacrificial layers on the (001) TiO_2_ substrate.

### Release and transfer of freestanding TiO_2_ NMs

To release the freestanding TiO_2_ NM from the VO_2_/TiO_2_ substrates, the surface of the grown heterostructure was adhered onto a rigid supporting layer (e.g., polydimethylsiloxane, thermal release tape (Haeun Chemtec, RP70N5)). The structure was immersed in a dilute H_2_O_2_ solution until the sacrificial VO_2_ layer was completely dissolved. To transfer the released TiO_2_ NM to other substrates, TiO_2_ NM was placed onto an oxide-coated silicon substrate and exposed to an appropriate temperature. Finally, the freestanding NM remained on the silicon substrate after peeling off the rigid supporting layer slowly. The structure was immersed in a dilute 10% H_2_O_2_ solution (i.e., 50 ml of 35% H_2_O_2_ + 150 ml of H_2_O) at room temperature until the sacrificial VO_2_ layer was completely dissolved, with the freestanding NM left on the rigid supporting layer. After dissolving the VO_2_ layers in H_2_O_2_, H_2_O residue on the released TiO_2_ surface was evaporated in a vacuum desiccator for 10 min. To transfer the released TiO_2_ NM to other substrates (such as thermally grown SiO_2_-coated silicon), the TiO_2_ NM/rigid supporting layer was attached to a oxide-coated silicon substrate and was heated to 60–80 °C for 10 min. Finally, the freestanding NM remained on the silicon substrate after peeling off the rigid supporting layer slowly.

### SEM, EBSD, and AFM measurements

OM images were recorded using BX53M (Olympus, Tokyo, Japan) microscope, equipped with an objective MPlanFL N (Olympus) and i-solution IMT cam CCD camera. The SEM and EBSD measurements were made using an XL30SFEG and FEI Helios Nanolab 650 equipped with an EBSD detector, respectively. The EBSD pattern was acquired by an EDAX Hikari EBSD camera while the sample was tilted at 70° and scanned with an electron beam of 25 nA at 20 kV. The measured data were analyzed using TSL OIM Analysis7. The AFM images were obtained using a VEECO Dimension 3100 in tapping mode.

### X-ray diffraction (XRD) and X-ray absorption spectroscopy (XAS)

High-resolution x-ray scattering was performed by using synchrotron radiation at the 3D XRS ($\lambda$ ~ 0.12398 nm, energy ~10 keV at Si (111)) beamline of Pohang Light Source-II (PLS-II, Pohang, Republic of Korea), and using an in-house HRXRD (Bruker Discover 8 X-ray diffractometer) with Cu K_α1_ radiation ($\lambda$ ~ 0.15406 nm). The detailed information on in-plane and out-of-plane lattice parameters and strain states of each films and NM was obtained by using both symmetric 2θ-ω scan and asymmetric RSM around the (112) reflection. X-ray absorption spectroscopy (XAS) was performed using the 2 A MS beamline at PLS-II. The total electron yield mode with an energy resolution of ~0.1 eV was used for measurement at a base pressure of 5 × 10^−10^ Torr in the analysis chamber by measuring the sample current (I_1_) divided by the beam current (I_0_) to remove the variation of the beam intensity.

### Scanning transmission electron microscope (STEM)

Two sample types were prepared for plane-view STEM imaging of transferred TiO_2_ NM on a carbon TEM grid and cross-sectional STEM imaging of transferred TiO_2_ NM and VO_2_/TiO_2_ hetero-NM on SiO_2_/Si. For the plane-view observation, the TiO_2_ film was directly transferred onto the carbon TEM grid; TiO_2_/VO_2_/TiO_2_ epitaxial heterostructure was physically attached to the PDMS with the carbon TEM grid and immersed in a dilute H_2_O_2_ solution. After selective oxidation and dissolution of the VO_2_ sacrificial layer, the single-crystal TiO_2_ NM was naturally released and attached to the carbon TEM grid (Supplementary Fig. 4). For the cross-sectional observation, the transferred TiO_2_ NM and VO_2_/TiO_2_ hetero-NM on SiO_2_/Si substrates were prepared by a focused ion beam (FIB) system (Helios G3, FEI), in which the samples were thinned by a Ga ion beam. The atomic structures were observed using a STEM (JEOL ARM 200 F, JEOL Ltd., Japan) with a fifth-order aberration corrector (ASCOR, CEOS GmbH, Heidelberg, Germany); the probe diameter and convergence angle of the beam were ~0.7 Å and ~27 mrad under an acceleration voltage of 200 kV, respectively. The collection semi-angles of the detectors for HAADF imaging were 54–210 mrad, and those for ABF imaging were 8–16 mrad to detect light elements (i.e., oxygen). The obtained STEM images were local difference filtered to reduce background noise (HREM Research Inc., Japan).

### Resistivity measurements

The resistivity (*ρ*) was measured in the van der Pauw geometry during heating and cooling from 260 to 380 K by using a chamber probe station equipped with a Hall measurement system and a temperature control system. The resistivity change over the MIT ($\Delta \rho /\rho$) was defined as $\Delta \rho /\rho =({\rho }_{{T}_{{{{{\mathrm{MIT}}}}}}-15K}-{\rho }_{{T}_{{{{{\mathrm{MIT}}}}}}+15K})/{\rho }_{{T}_{{{{{\mathrm{MIT}}}}}}+15K}$. The fitting of the derivative of log_10_ ($\rho$) as a function of temperature (K) was performed based on the Gaussian function. *T*_h_ and *T*_c_ were then defined at the peak position of the Gaussian and the transition width was calculated from the difference ($\triangle H$ = *T*_h_ − *T*_c_). The transition sharpness $\triangle {T}_{{{{\mathrm{h}}}}}$ and $\triangle {T}_{{{{\mathrm{c}}}}}$ was estimated from the full-width at half-maximum of the Gaussian peak.

## Supplementary information

Supplementary Information

Peer Review File

## Data Availability

All relevant data within the article are available from the corresponding authors on reasonable request.
