# Supplementary material for: Heterogeneous integration of single-crystalline rutile nanomembranes with steep phase transition on silicon substrates
Source: Nat Commun. 2021 Aug 18;12:5019. doi: 10.1038/s41467-021-24740-2 (PMC8373986; doi:10.1038/s41467-021-24740-2)
Supplement: Supplementary file 1 — Supplementary Information [file 41467_2021_24740_MOESM1_ESM.pdf]

Supplementary information for

Heterogeneous integration of single-crystalline rutile nanomembranes with steep phase transition on silicon substrates

Dong Kyu Lee[1),+], Yunkyu Park[1),+], Hyeji Sim[1),+], Jinheon Park[1)], Younghak Kim[2)], Gi-Yeop Kim[1)], Chang-Beom Eom[3)], Si-Young Choi[1),#], Junwoo Son[1),*]

1) Department of Materials Science and Engineering, Pohang University of Science and Technology (POSTECH), Pohang 37673, Republic of Korea

2) Pohang Accelerator Laboratory, Pohang 37673, Republic of Korea

3) Department of Materials Science and Engineering, University of Wisconsin-Madison, Madison, Wisconsin 53706, United States

[+] These authors contributed equally to this work

[*] jwson@postech.ac.kr ; [#] youngchoi@postech.ac.kr

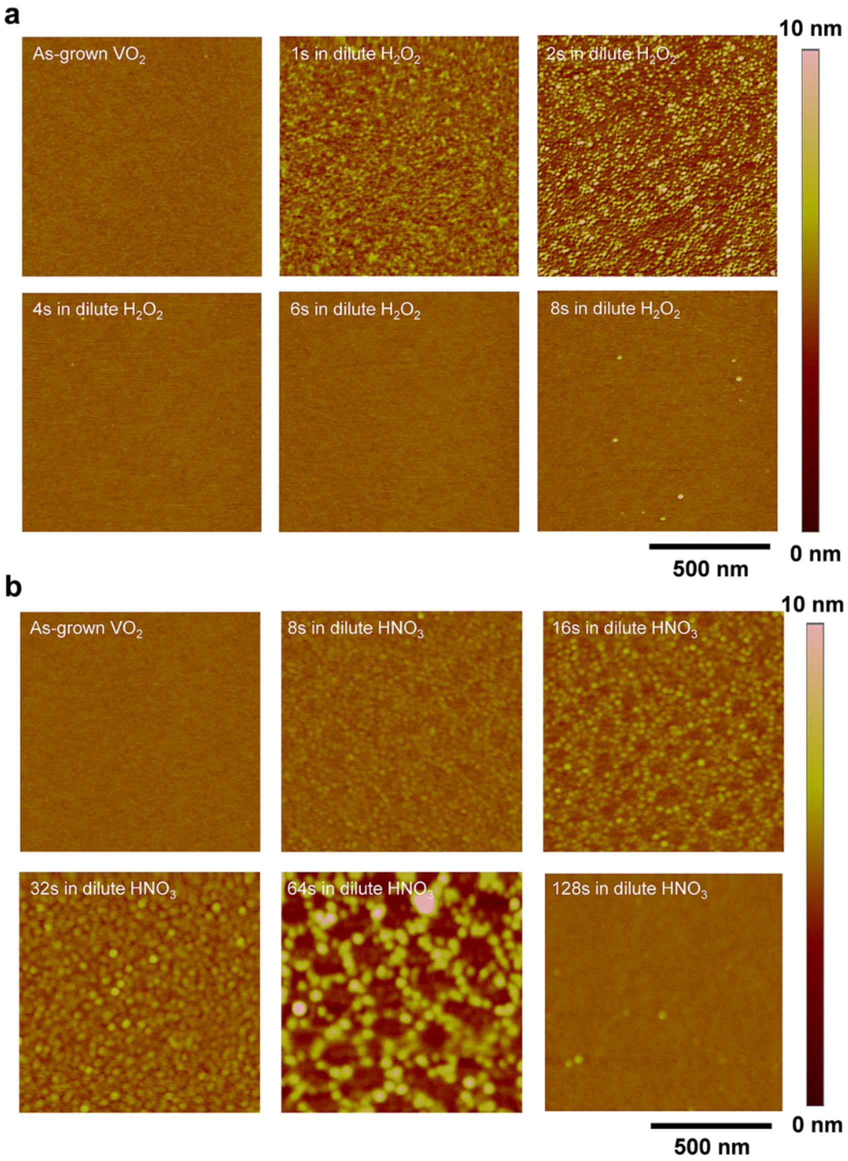

Supplementary Figure 1 | **a,** AFM image of 10-nm-thick VO_2 films as a function of etching time (0 ~ 8 sec) after immersion into 10 % dilute H_2O_2 solution (pH ~ 5.3). After 4 seconds, the surface was as clean as the TiO_2 surface before VO_2 growth (see **Fig. S2**), which indicates that the H_2O_2 solutions completely removed VO_2 films free of any residues on the surface of TiO_2 substrates within 4 seconds. **b,** AFM image of 10-nm-thick VO_2 films as a function of etching time (0 ~ 128 sec) after immersion into 10 % dilute HNO_3 solution (pH ~ 2). The residue of VO_2 films still remained even after 64 seconds despite more acidic condition. High etching capability of H_2O_2 on VO_2 was confirmed by direct comparison with that of HNO_3 with same concentration.

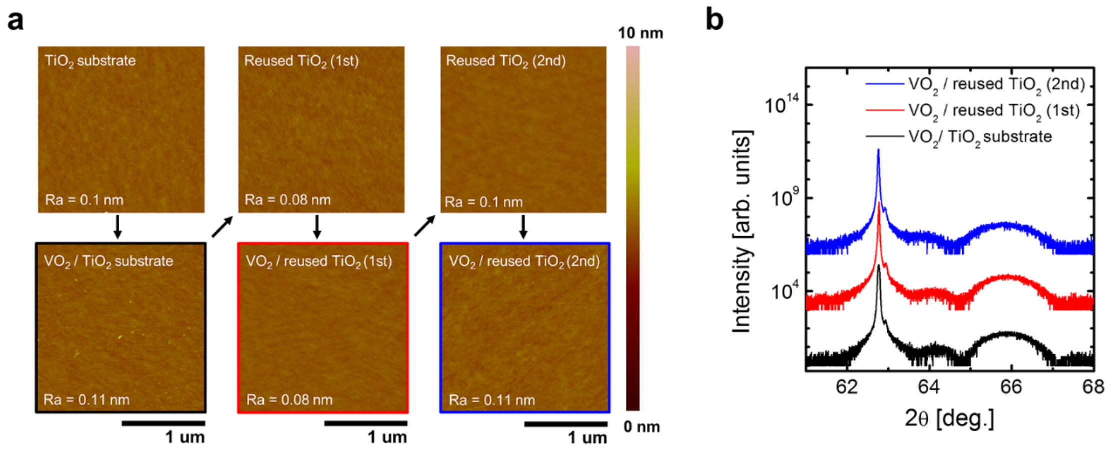

Supplementary Figure 2 | TiO_2 substrate reusability test for the production of TiO_2 nanomembrane. The epitaxial growth and subsequent etching of VO_2 sacrificial layers on TiO_2 substrates were repeated to confirm intact surface morphology of TiO_2 substrates after the removal of VO_2 layer by H_2O_2. **a,** the AFM image of TiO_2 substrates and VO_2 films on TiO_2 substrates before the removal of VO_2 layer (left), after 1st removal of VO_2 layer (center) and after 2nd removal of VO_2 layer in dilute H_2O_2 solution (right). Sequential growth and removal of VO_2 layer by dilute H_2O_2 solution preserve the atomically flat surface topography of TiO_2 substrates (R_a ~ 0.1 nm) without any residue. **b**, Symmetrical XRD $2\theta - \omega$ scans of VO_2 films on TiO_2 substrates on pristine and reused TiO_2 substrates. The high-quality VO_2 sacrificial layers were epitaxially grown on reused TiO_2 substrates after the removal of VO_2 sacrificial layer by dilute H_2O_2 solution, which shows the possible synthesis of TiO_2 nanomembranes on VO_2/reused TiO_2 substrates.

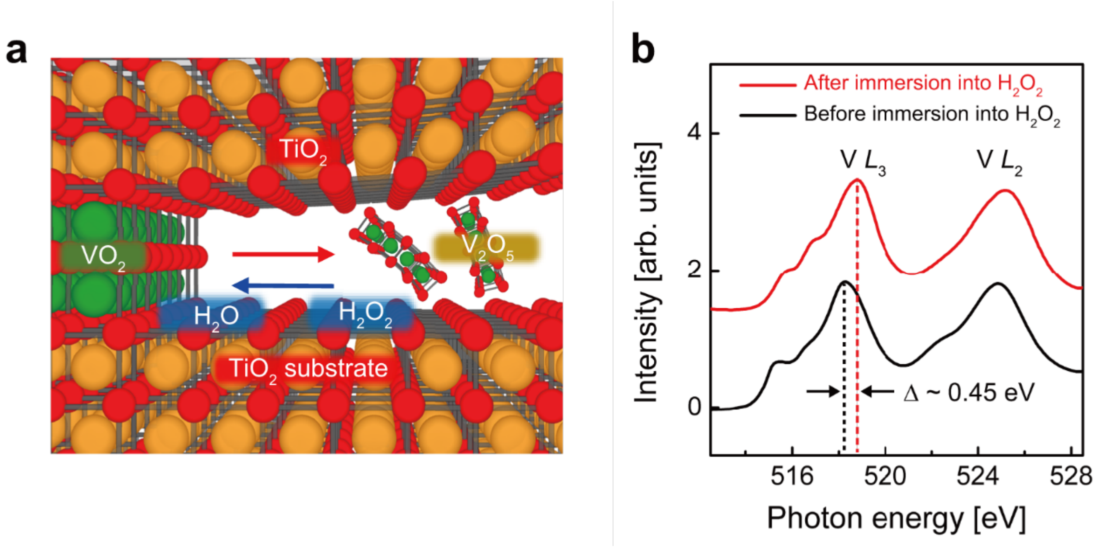

Supplementary Figure 3 | **a,** The illustration of oxidation and dissolution in the VO_2 sacrificial layer by the chemical reaction with H_2O_2. **b,** XAS at the V $L_{2,3}$-edge before (black) and after (red) immersing epitaxial VO_2 films in dilute H_2O_2 solution, which experimentally confirm the oxidation of VO_2 sacrificial layer by H_2O_2. The V L_3-edge peak of the films (black line) shifted to a higher photon energy about 0.45eV (red line), and this shift is attributed to a change in the oxidation state from V^{4+} to V^{5+} owing to the decrease of the electron occupancy.

Unlike the etching of VO_2 films to use strong acids (e.g., HNO_3, *J. Colloid. Interf. Sci.* 512, 529-535 (2018)) or alkalis (e.g., NaOH, *ACS Appl. Mater. Interfaces* 8, 14863 (2016)), strikingly, in our study, H_2O_2 as a strong oxidant appears to result in rapid destabilization of VO_2 lattices even under a mild pH (~ 5.3) condition. Two feasible thermodynamic reactions, ($2\,VO_2(s) + H_2O_2(l) \leftrightarrow V_2O_5(s) + H_2O(l)$ or $2\,VO_2(s) + H_2O_2(l) \leftrightarrow V_2O_5 \cdot H_2O(s)$), are likely to occur during the immersion of VO_2 films into H_2O_2 solution (*Chem. Mater.* 3, 578-593 (1991), *Environ. Sci. Technol.* 52, 2178-2185 (2018)), and thermodynamic calculations using these reaction yielded a Gibbs free energy with a significant driving force toward the right direction ($\Delta G = -224.21$ kJ/mol at equation (1) and $\Delta G = -355.36$ kJ/mol at equation (2)); VO_2 films are spontaneously oxidized by H_2O_2 and transformed to V_2O_5 and/or water-soluble $V_2O_5 \cdot H_2O(s)$ gels with a layered van der Waals (vdW) structure along the c-axis; these layered crystals with weak bonding are exfoliated and dispersed in the solution.

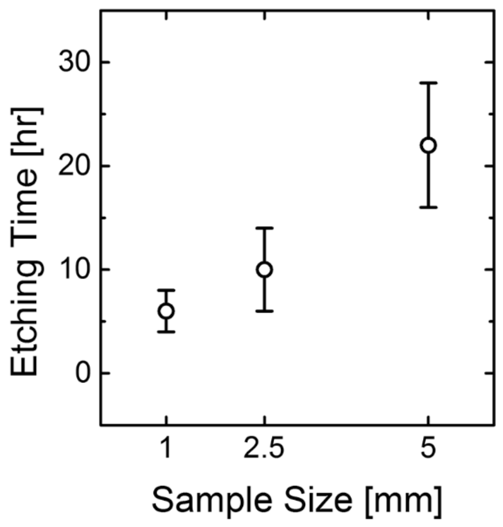

Supplementary Figure 4 | The dissolution (or release) time of TiO_2 NMs attached on supporting layers from as-grown TiO_2 (70 nm)/VO_2 (15 nm)/TiO_2 heterostructures with various sample size (1 mm × 1 mm, 2.5 mm × 2.5 mm, 5 mm × 5 mm). Error bars are included in each point.

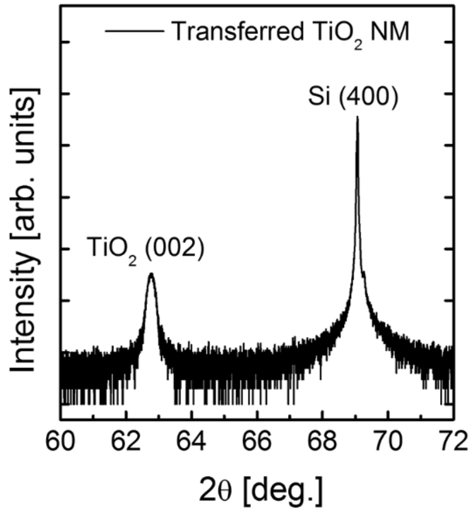

Supplementary Figure 5 | X-ray diffraction 2θ-ω scan of transferred TiO_2 NM on silicon substrate using in-house HRXRD with Cu $K_{\alpha 1}$ radiation

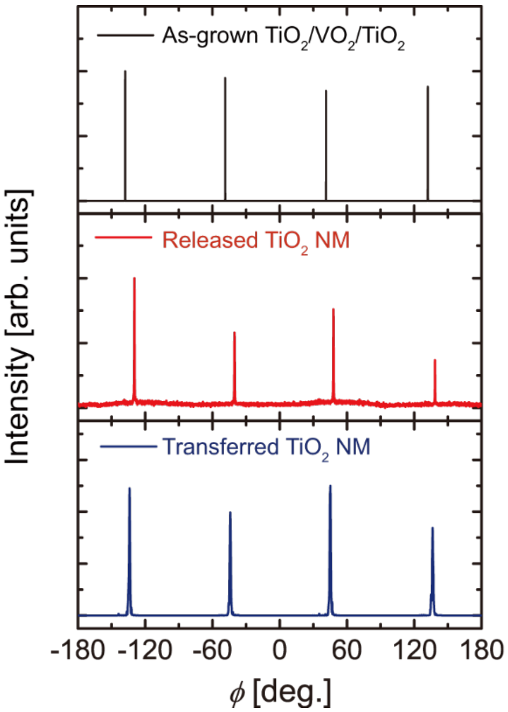

Supplementary Figure 6 | Asymmetric ϕ scans around (112) reflection of as-grown 70 nm-thick TiO_2 epitaxial films and TiO_2 single-crystal NM released on PDMS and transferred on Si substrates. The high-resolution X-ray diffraction measurements shows single-crystallinity with no in-plane rotation in released and transferred TiO_2 NM.

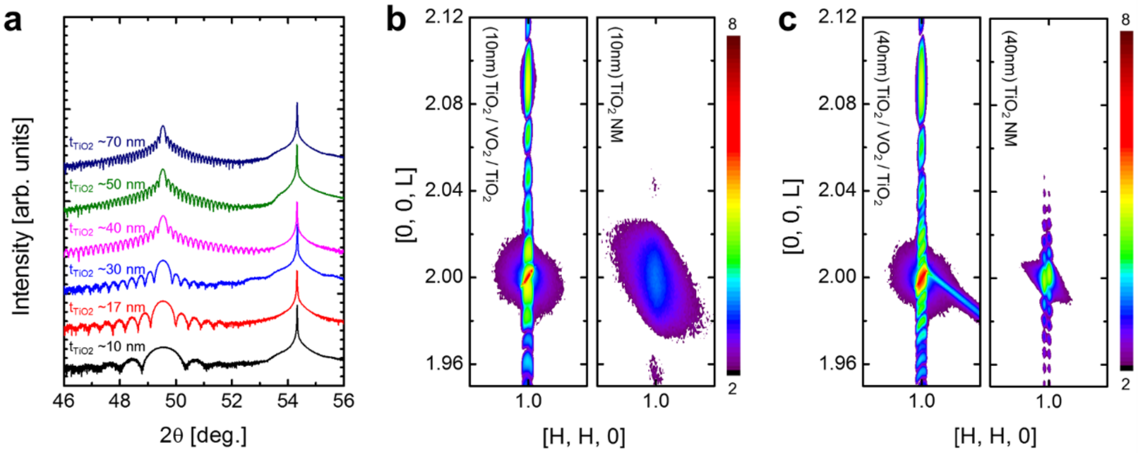

Supplementary Figure 7 | High crystalline quality of transferred TiO_2 single-crystal nanomembranes (NM) with various thickness. **a,** symmetrical 2θ – ω scans of transferred TiO_2 single-crystal nanomembranes with a range of thickness (10 nm ~ 70 nm). **b,** reciprocal space mapping around (112) reflection of as-grown 10 nm-thick TiO_2 epitaxial films on VO_2/TiO_2 substrates (left) and 10 nm-thick TiO_2 single-crystal NM transferred on Si substrates (right). **c,** reciprocal space mapping around (112) reflection of as-grown 40 nm-thick TiO_2 epitaxial films on VO_2/TiO_2 substrates (left) and 40 nm-thick TiO_2 single-crystal NM transferred on Si substrates (right). Since VO_2 sacrificial layers are coherently grown on the TiO_2 substrates with identical H, strain-free epitaxial TiO_2 layers with various thickness are coherently grown on VO_2/TiO_2 substrates; these strain-free TiO_2 layers with precisely controlled thickness are released and transferred on Si substrate without the modification of lattice parameters and crystallinity.

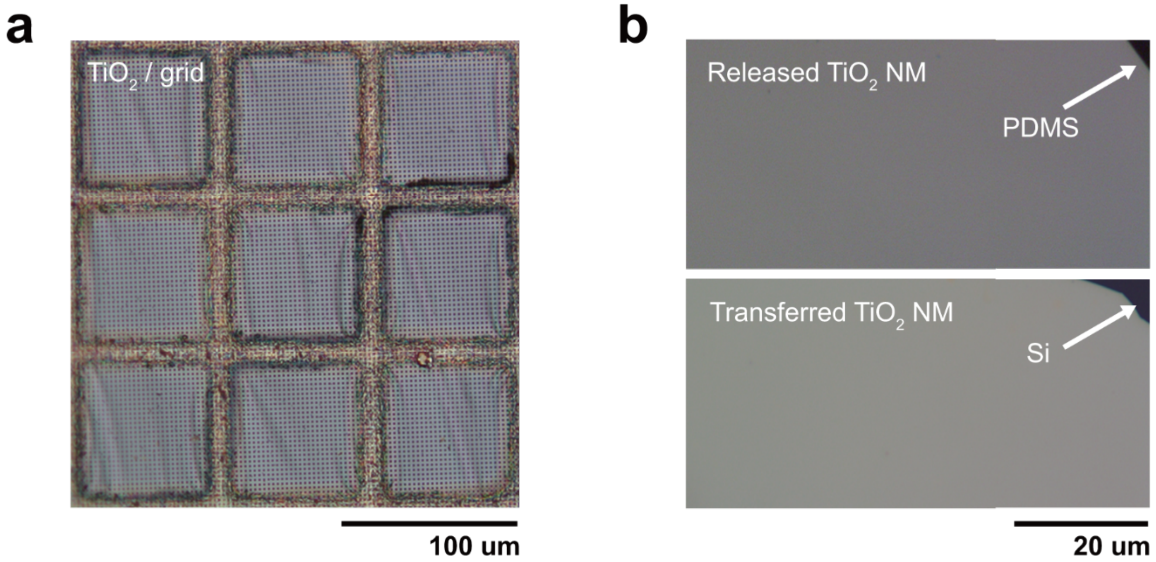

Supplementary Figure 8 | a. OM image of transferred TiO_2 NM with natural wrinkle on the carbon TEM grid. **b.** SEM image of released and transferred TiO_2 NM, which confirms uniform and flat surface without surface crack or residues.

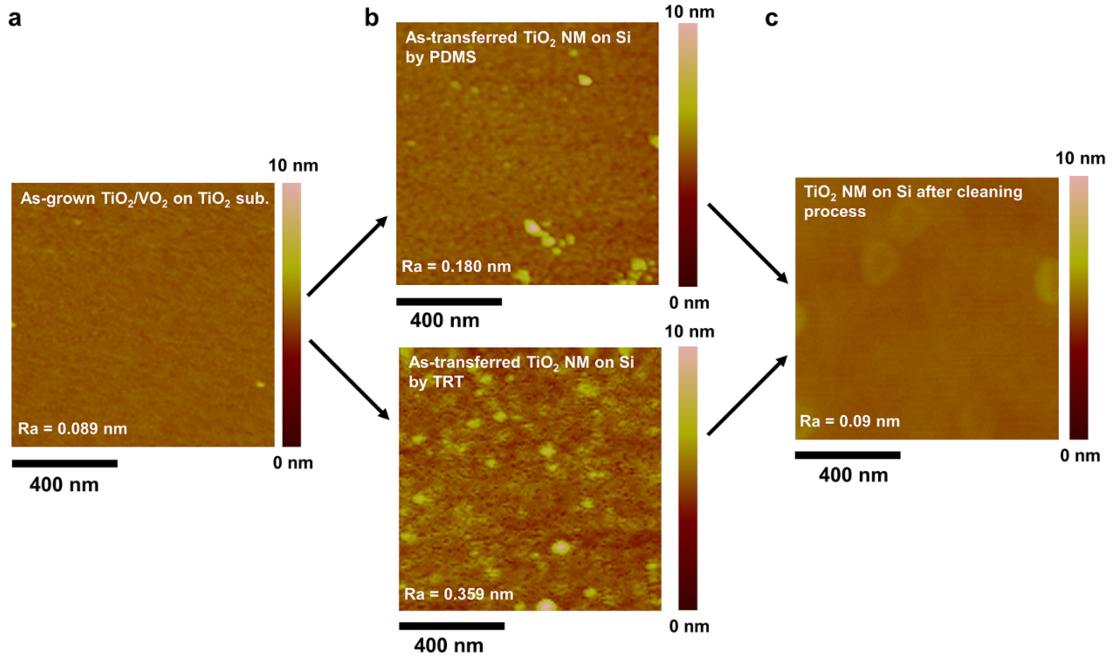

Supplementary Figure 9 | Atomic force microscopy image of **a.** "as-grown" TiO_2/VO_2 epitaxial films on the TiO_2 single crystal substrates, **b.** "as-transferred" TiO_2 NM on silicon substrate, **c.** transferred TiO_2 NM on silicon substrate "after our RCA cleaning process". The surface of the TiO_2 NM on Si is as good as that of as-grown TiO_2 epitaxial films on VO_2/TiO_2.

Below is our detailed procedure for the removal of residues on the surfaces of TiO_2 NM

Step 1: Transferred TiO_2 NM/Si was immersed in acetone (30 minutes), isopropyl alcohol (10 minutes), and de-ionized water (10 minutes) to remove organic residues.

Step 2: To strengthen the bonding between the TiO_2 NM and Si substrate and prevent delamination of the TiO_2 NM during subsequent cleaning process, transferred TiO_2 NM/Si was annealed at 500 °C for 3 hours.

Step 3: After thermal annealing, transferred TiO_2 NM/Si was immersed in the solution of 5:1:1 H_2O / NH_4OH / H_2O_2 at 80 °C for 10 minutes to removing residual organic contaminants.

Step 4: And then, residual metal contaminates was removed by using a solution of 5:1:1 H_2O / HCl / H_2O_2 at 80 °C for 10 minutes.

Step 5: Repeat 'Step 3' before growing VO_2 epitaxial film on TiO_2 NM.

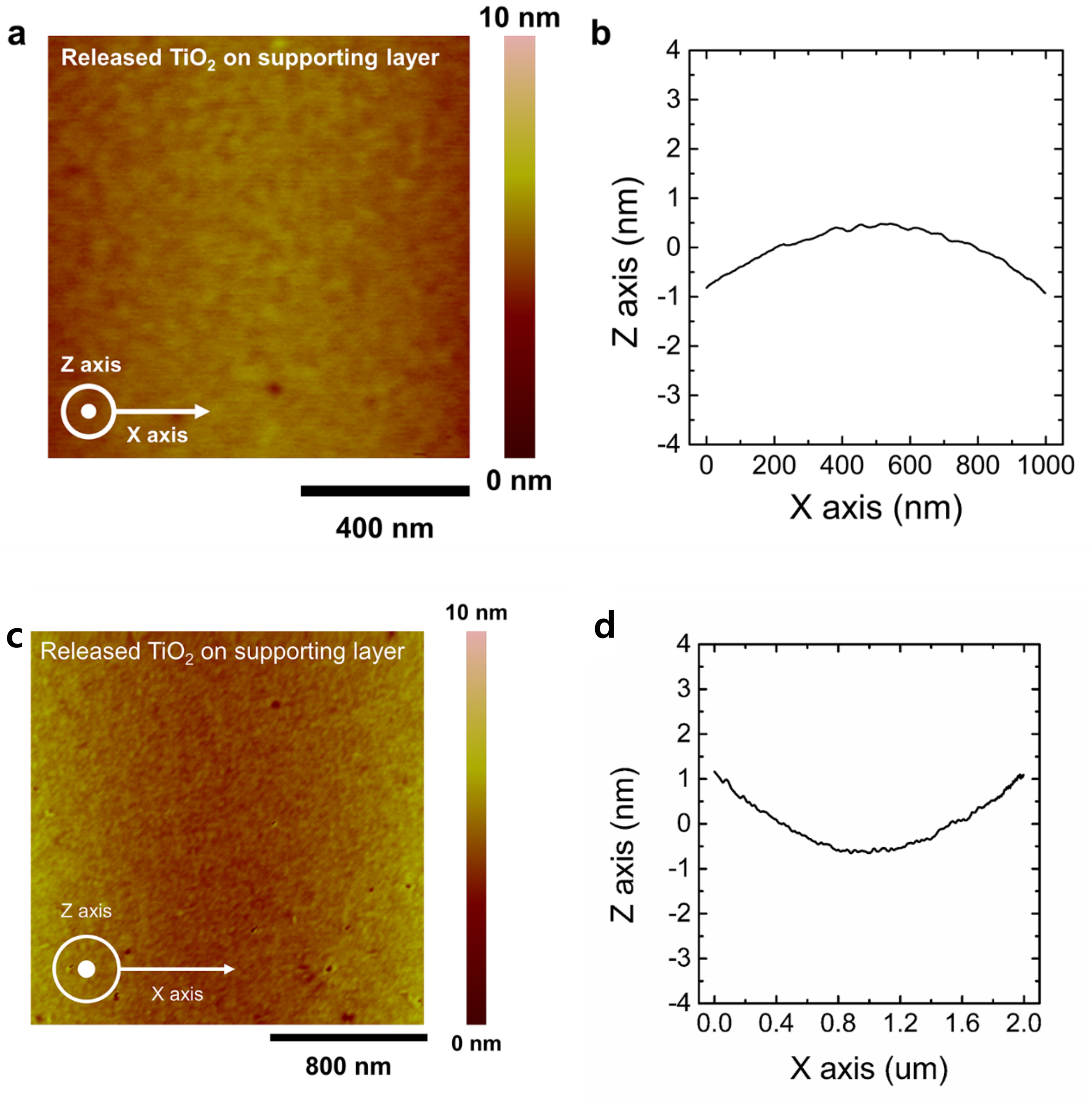

Supplementary Figure 10 | a. Atomic force microscopy flattened image and **b.** depth profile along the x-axis direction of released TiO_2 NM on supporting layer. Except height modulation by the slight ourward bending due to the elastic supporting layer, no VO_2 residue or any damages was observed at the surface of TiO_2 NM after H_2O_2 treatment. **c, d,** Inward bending of the elastic supporting layer in the different spot.

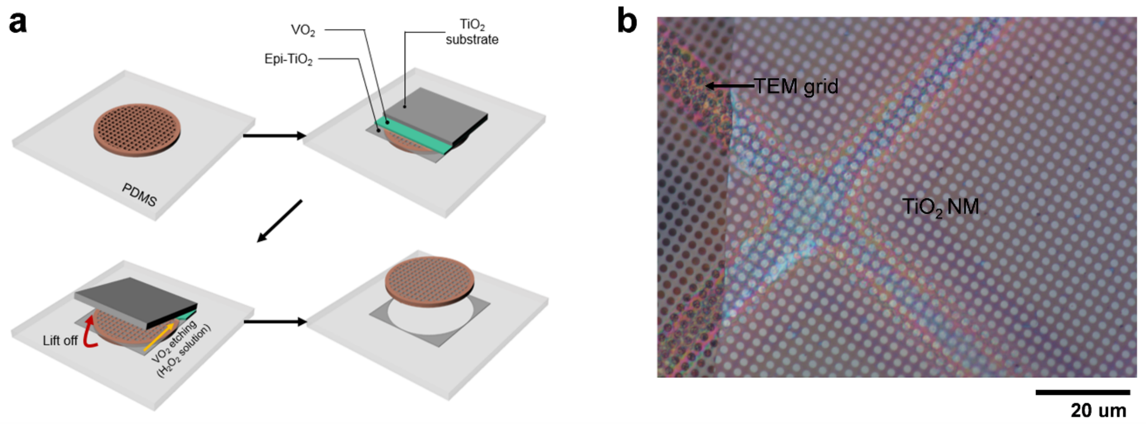

Supplementary Figure 11 | a, A fabrication process for plain-view observation of TiO_2 NM on carbon coated TEM grid. At first, carbon TEM grid was attached on PDMS rigid supporting layer. Then, TiO_2 / VO_2/ TiO_2 epitaxial heterostructure was physically attached to the PDMS with carbon TEM grid and immersed into dilute H_2O_2 solution. After selective oxidation and dissolution of VO_2 sacrificial layer, single-crystal TiO_2 NM was naturally released and attached on the carbon TEM grid. Finally, TEM grid with TiO_2 NM was detached from PDMS rigid supporting layer. **b,** magnified optical microscope image of single-crystal TiO_2 NM on carbon TEM grid.

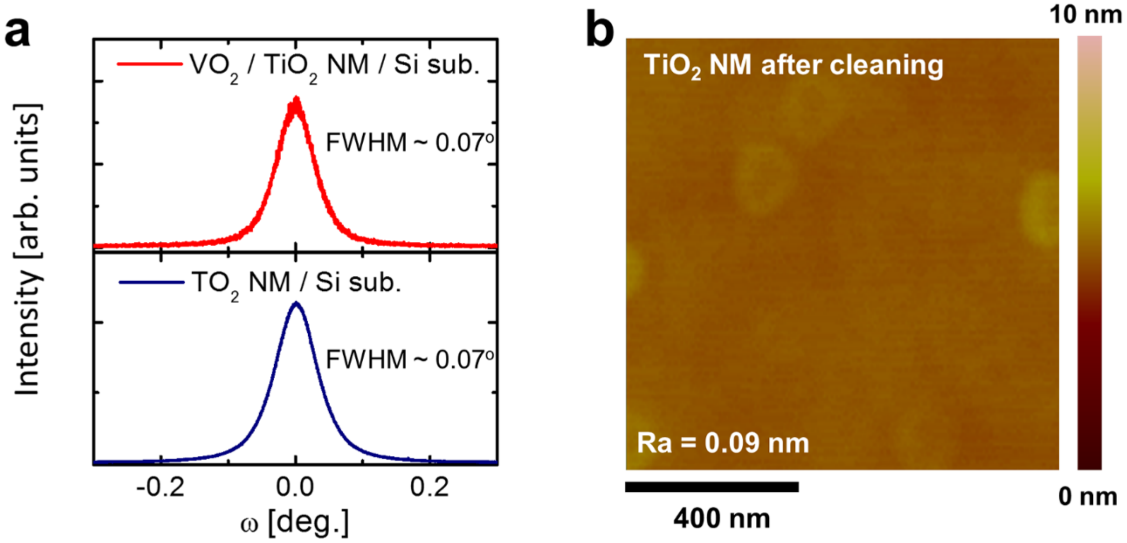

Supplementary Figure 12 | a, Rocking curve of (002) VO_2 peak and (002) TiO_2 peak in the VO_2/TiO_2 NM on the Si substrate. FWHM is approximately 0.07°, which indicates all oxide films consisted of VO_2 and TiO_2 NM have high crystal quality. **b,** AFM image of VO_2/TiO_2 NM verifies very clean surface.

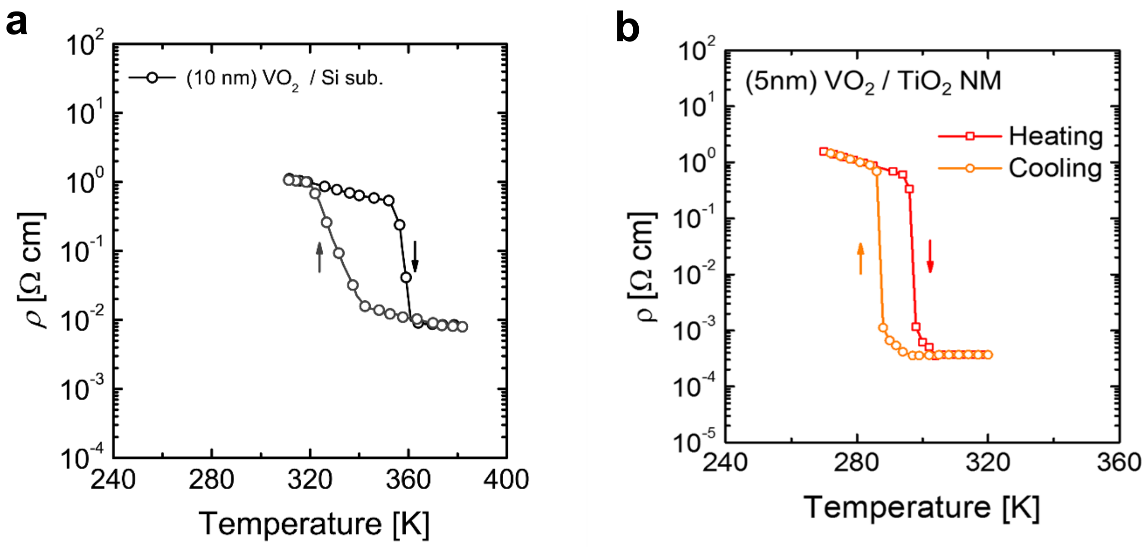

Supplementary Figure 13 | Resistivity as function of temperature of **a,** 10 nm-thick VO_2 films directly grown on the SiO_2 / Si substrate and b, 5 nm-thick VO_2 films on TiO_2 NM/Si. Temperature-dependent metal-insulator transition is significantly degraded compared to 10 nm-thick VO_2/ TiO_2 NM / Si substrate (see **Fig. 5a**), probably due to interfacial reaction and the formation of defective layer (**a**). Sharp metal-insulator transition characteristic with high resistivity ratio ($\Delta\rho/\rho > 10^3$) was observed even in 5 nm-thick VO_2 films grown on TiO_2 NM.

Method	Thickness	T_{MIT}	$\rho_{T_{MIT}-15K}$	$\rho_{T_{MIT}+15K}$	$\Delta\rho/\rho$	Reference
ALD	4nm	356K	3	0.9	2.33	*Adv. Funct. Mater.* **25**, 679-686 (2015).
	8nm	340K	0.03	0.001	29	*Adv. Funct. Mater.* **25**, 679-686 (2015).
	14nm	360K	0.003	0.00003	99	*Adv. Funct. Mater.* **25**, 679-686 (2015).
	28nm	325K	0.004	0.00003	132	*Adv. Funct. Mater.* **25**, 679-686 (2015).
	30nm	355K	6	0.03	199	*ECS J. Solid State Sci. Technol.* **1**, P169 (2012).
PLD	80nm	337K	6.4	0.024	265	*Thin Solid Films* **632**, 119-127 (2017).
	19.5nm	318K	0.6	0.02	29	*Acta Mater.* **137**, 12-21 (2017)
	21.91nm	325K	0.4	0.004	99	*Acta Mater.* **137**, 12-21 (2017)
	50nm	340K	0.8	0.005	159	*J. Appl. Phys.* **118**, 055301 (2015).
Sol-Gel	200nm	337K	0.016	0.00001	1599	*J. Appl. Phys.* **70**, 443-452 (1991)
Sputter	210nm	338K	0.32	0.0015	212.33	*J. Appl. Phys.* **114**, 244301 (2013).
	210nm	328K	0.357	0.000735	484.71	*J. Phys. D Appl. Phys.* **47**, 455304 (2014).
	100nm	336K	0.5	0.025	19	*J. Appl. Phys.* **108**, 073708 (2010).
	300nm	335K	0.6	0.0195	29.76	*J. Mater. Chem. C* **6**, 1722-1730 (2018).
	10nm	328K	0.095	0.018	4.27	*Appl. Phys. Lett.* **98**, 192113 (2011).
	30nm	328K	0.07	0.011	5.36	*Appl. Phys. Lett.* **98**, 192113 (2011).
	50nm	328K	0.06	0.0075	7	*Appl. Phys. Lett.* **98**, 192113 (2011).
	70nm	325K	0.07	0.0075	8.33	*Appl. Phys. Lett.* **98**, 192113 (2011).
	90nm	325K	0.037	0.0018	19.55	*Appl. Phys. Lett.* **98**, 192113 (2011).
	110nm	325K	0.035	0.00095	35.84	*Appl. Phys. Lett.* **98**, 192113 (2011).
	130nm	325K	0.02	0.00065	29.76	*Appl. Phys. Lett.* **98**, 192113 (2011).
	190nm	333K	0.035	0.00065	52.84	*Appl. Phys. Lett.* **98**, 192113 (2011).
	210nm	333K	0.037	0.0006	60.66	*Appl. Phys. Lett.* **98**, 192113 (2011).

Supplementary Table 1 | Metal-insulator transition properties of VO_2 thin films on Si (or oxide-coated Si) substrates from previous literatures. These data allow for a direct comparison with those from our VO_2 films on TiO_2 NM templated Si substrate in **Fig. 5c.**

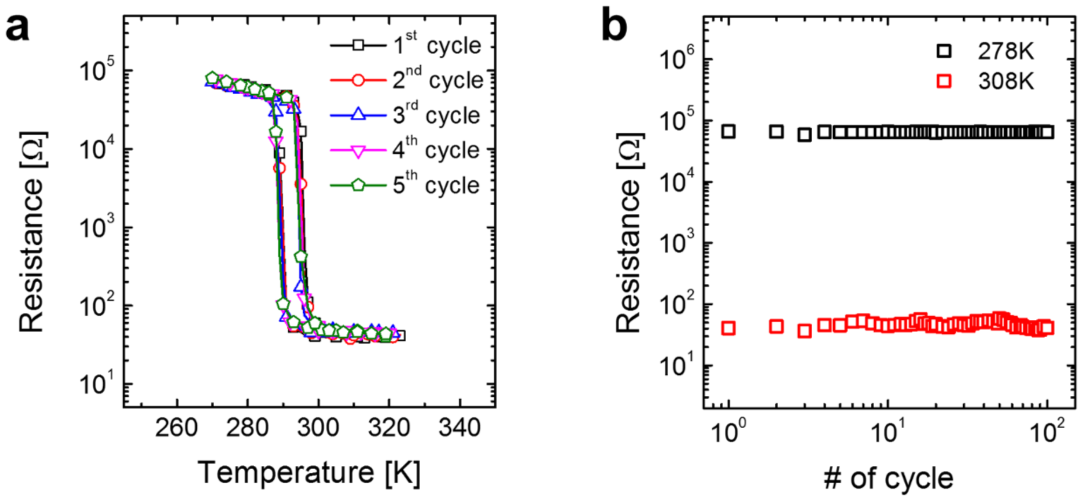

Supplementary Figure 14 | a, Repeated measurement of temperature-dependent resistance from 270 K to 320 K during 5 cycles. **b,** thermal cycling at 278 K and 308 K until 100 cycles in the 10 nm-thick VO_2 / 70 nm-thick TiO_2 NM / Si.

Steep sheet resistance modulation with metal-insulator transition across $T_{MI} \sim 296$ K ($\Delta\rho/\rho \sim 10^3$) were observed in 10 nm-thick VO_2 on TiO_2 NM/Si more than 100 cycles. No drift was observed during the multiple cycle of thermal switching of metal-insulator transition in VO_2 on TiO_2 NM/Si

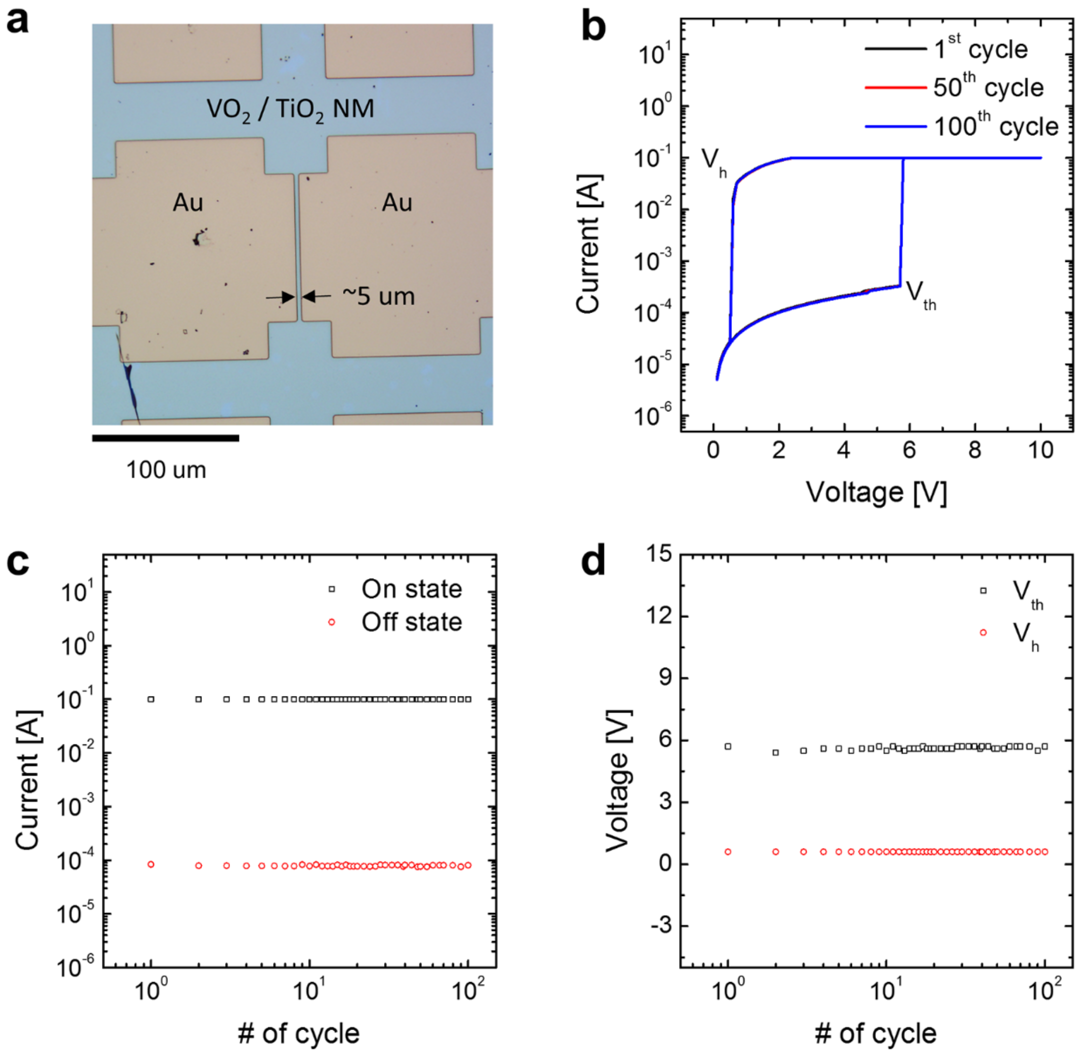

Supplementary Figure 15 | a, Plane view of two-terminal threshold switch with single crystalline VO_2/TiO_2 NM on Si obtained by optical microscopy. **b,** The I-V characteristic of two-terminal single crystal VO_2 device consisted of Au/Ti electrodes on the VO_2/TiO_2 NM with 5 um channel length. **c,** The DC endurance of the singe crystal VO_2 device, which showed stable resistivity change. **d,** Homogeneous V_{th} and V_h changes during DC bias endurance more than 100 cycles.

Two terminal devices with "single-crystalline" VO_2 integrated on Si showed high reliable selectivity of on and off states ($I_{on}/I_{off} > 10^3$) more than 100 cycles, which shows excellent the endurance of I_{on}/I_{off} ratio, V_{th} and V_h during DC bias sweep

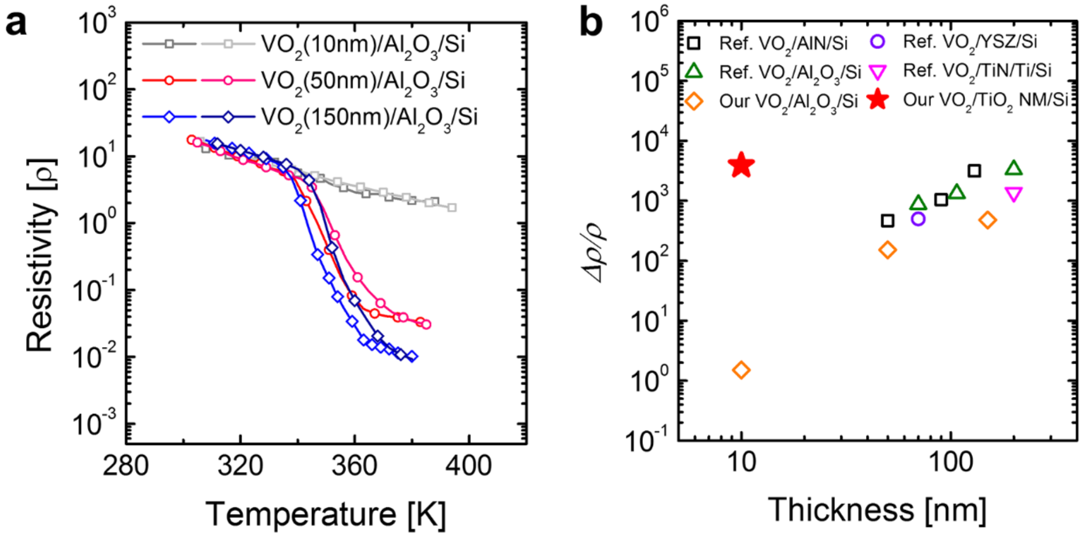

Supplementary Figure 16 | a, Temperature-dependent resistivity modulation near T_{MIT} in 10-nm-thick VO_2 film (gray line), 50-nm-thick VO_2 film (red line) and 150-nm-thick VO_2 film (blue line) grown on Al_2O_3 buffered Si substrate. **b,** Benchmark of resistivity ratio $\boldsymbol{\Delta\rho/\rho = (\rho_{T_{MIT}-15K} - \rho_{T_{MIT}+15K})/\rho_{T_{MIT}+15K}}$ for VO_2 films on the buffered Si substrates across the MIT. For a direct comparison, all films were grown on the buffered Si substrates using various buffer layers: (Al_2O_3 buffer layer (green triangle) from ref. *Jpn. J. Appl. Phys.* **47**, 3067 (2008), *J. Phys. D: Appl. Phys.*, **47**, 455304 (2014), *J. Appl. Phys.* ***127**, 205303 (2020)*, AlN buffer layer (black square) from ref. *APL Mater.* 4, 026101 (2016), YSZ buffer layer (blue circle) from ref. *Appl. Phys. Lett.* 95, 111915 (2009), TiN/Ti buffer layer (pink inverted triangle) from ref. *J. Vac. Sci. Technol. A*, 32, 041502 (2014) and the Al_2O_3 buffer layer from Fig. S16a (orange diamond)). The $\boldsymbol{\Delta\rho/\rho}$ in 10-nm-thick "single-crystalline" VO_2 films on TiO_2 NM/Si ($\boldsymbol{\Delta\rho/\rho}$ ~ 3.3 × 10^3) shows more than 3 orders of magnitude higher than that in 10-nm-thick VO_2 films on Al_2O_3-buffered/Si substrate ($\boldsymbol{\Delta\rho/\rho_{10nm} \sim 1.50}$). In the case of VO_2 films on buffered Si, a further reduction in $\boldsymbol{\Delta\rho/\rho}$ was found to occur with decreasing film thickness, likely due to a number of interfacial defects and domain boundaries between buffer layer and VO_2 film

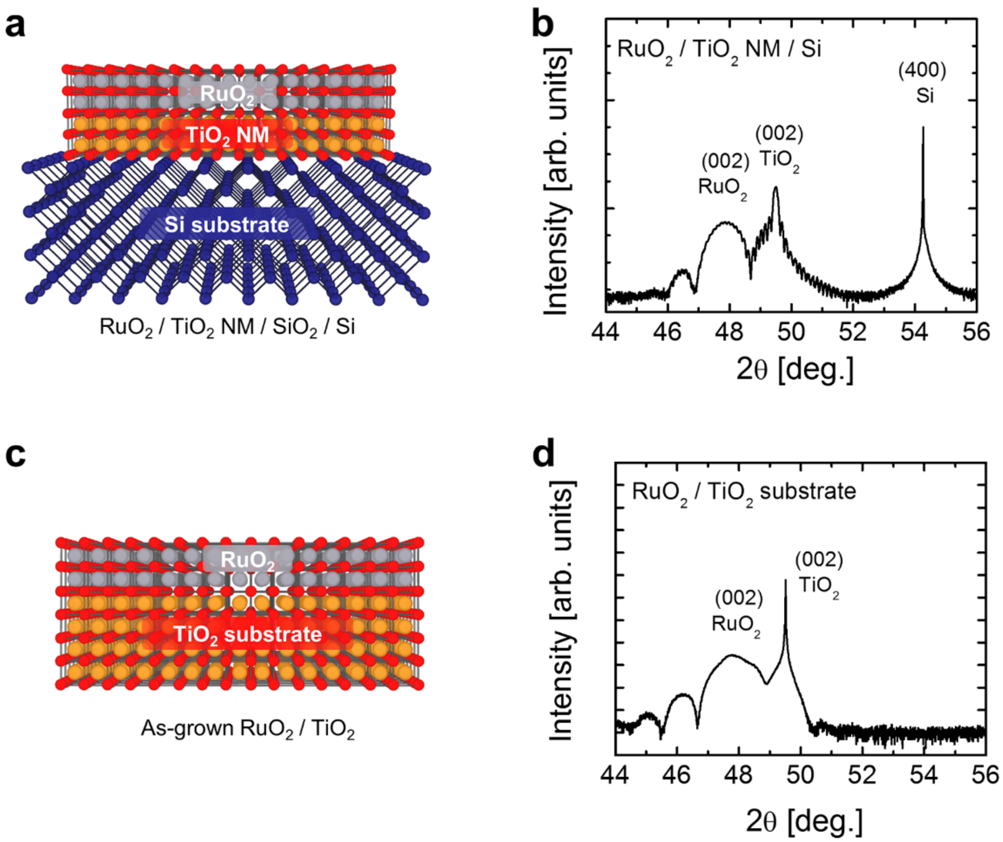

Supplementary Figure 17 | Schematic of RuO_2 film grown on the TiO_2 NM / Si substrate (**a**) and RuO_2 film grown on the (001) TiO_2 substrate (**c**). Corresponded symmetric XRD 2θ-ω scans of RuO_2 / TiO_2 NM / Si (**b**) and RuO_2 / (001) TiO_2 substrate (**d**). The location of (002) RuO_2 peak on TiO_2 NM/Si was almost identical that of epitaxial RuO_2 films grown on (001) TiO_2 substrates.

To generally demonstrate the heterogeneous integration of other rutile oxide single-crystalline layers on Si, we grew epitaxial RuO_2 films on TiO_2 NM/Si (**a**). After the cleaning process to remove the residue on transferred TiO_2 NM, 9-nm-thick RuO_2 thin films, instead of VO_2, were grown on TiO_2 NM/Si to realize RuO_2/TiO_2 epitaxial heterostructure integrated on Si substrates by pulsed laser deposition. As observed in symmetrical XRD 2θ-ω scans, the intense (002) RuO_2 peak appeared at ~ 2θ = 47.8°, along with peaks related to the TiO_2 template (~ 2θ = 49.5°) and Si substrates (~ 2θ = 54.3°) (**b**). The location of (002) RuO_2 peak on TiO_2 NM/Si (above figure in **b** was almost identical that of epitaxial RuO_2 films grown on (001) TiO_2 substrates (above figure in **d**); this result implicates that TiO_2 "single-crystalline" NM templates facilitate the formation of epitaxial RuO_2 films on Si substrates as well.

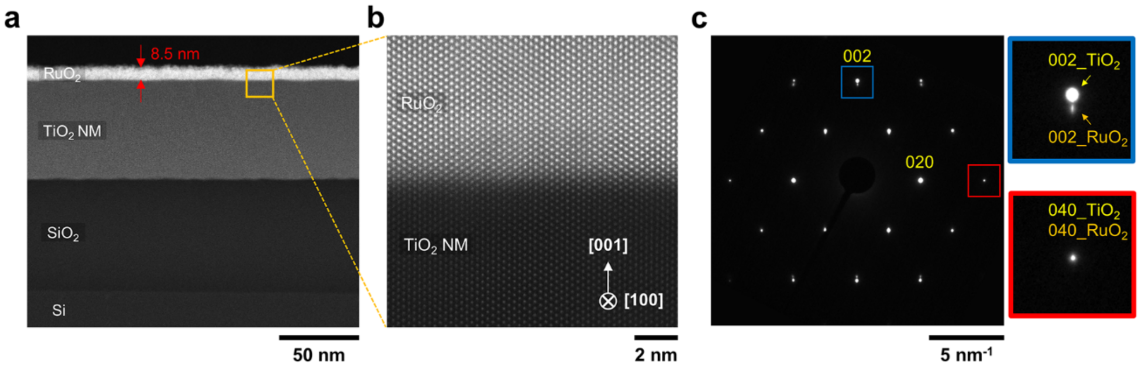

Supplementary Figure 18 | STEM analysis of RuO_2/TiO_2 NM on SiO_2/Si substrate. a, The cross sectional HAADF-STEM image of the heterogeneous RuO_2/TiO_2 NM on SiO_2/Si. The RuO_2 film appears much brighter than TiO_2 NM because the atomic number of Ru (Z_{Ru}=44) is much larger than that of Ti (Z_{Ti}=22). The low magnification image reveals that the RuO_2 thin films were uniformly deposited on TIO_2 NM with 8.5 nm thickness. The atomic-scale resolution image near the interface between RuO_2/TiO_2, indicated by yellow square in **a**, is shown in **b** (zone axis : [100] in both TiO_2 and RuO_2). The atomic columns of RuO_2 and TiO_2 are coherently matched at the interface with perfect rutile crystal structure; it means TiO_2 NM on SiO_2/Si allows the epitaxy growth of RuO_2 thin films despite the large lattice mismatch (~ 2%). **c,** SADP images on RuO_2/TiO_2 NM regions. The sharp diffraction spots shows both RuO_2 film and TiO_2 NM have high quality of rutile crystal structures. The out-of-plane direction diffraction spots (i.e., 002, indicated by blue square) are slightly separated due to lattice mismatch between RuO_2 and TiO_2, whereas the in-plane direction diffraction spots (i.e., 040, indicated by red square) are completely overlapped as a single spot, also confirming that epitaxy growth of RuO_2 thin films on TiO_2 NM. When comparing the lattice parameter c/a(=c/b) ratio calculated from the SADP images with the c/a ratio in the bulk states, there were differences by -0.98% and -4.48% in TiO_2 and RuO_2, respectively. The large decrease of c/a in RuO_2 indicates that the RuO_2 films are fully strained by biaxial tensile strain along the in-plane direction.

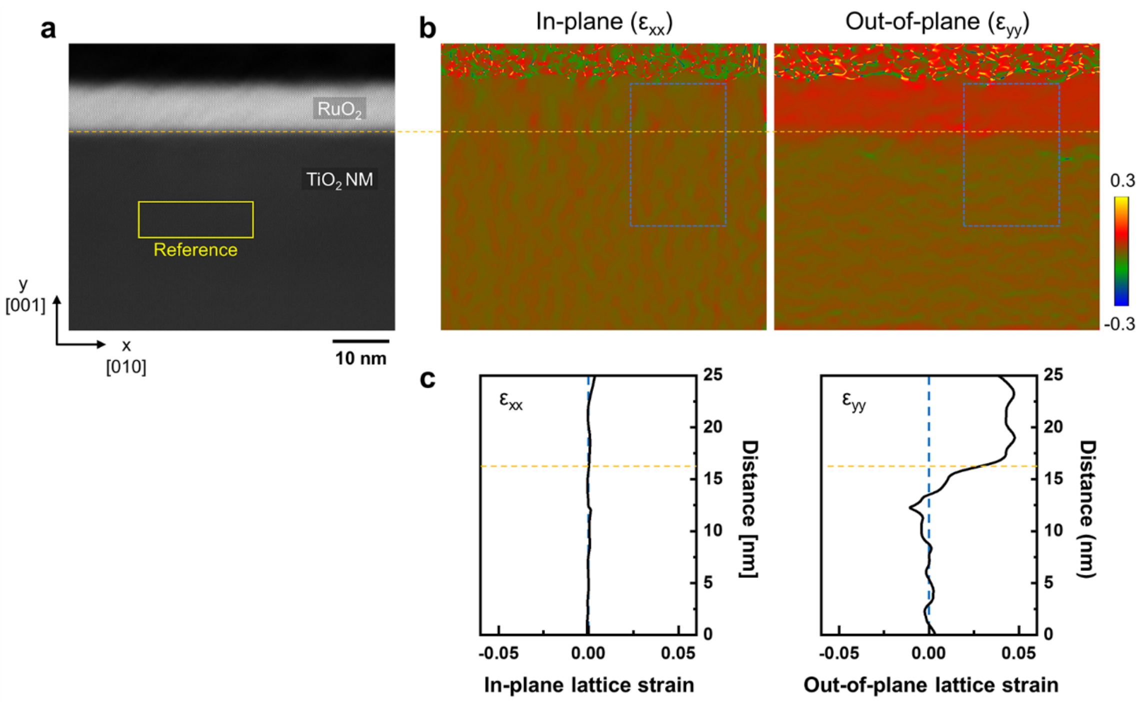

Supplementary Figure 19 | Local strain analysis of RuO_2/TiO_2 NM on Si substrate. a, HAADF-STEM image of RuO_2 thin films on the TiO_2 NM-template. **b,** In-plane (ε_{IP}) and out-of-plane (ε_{OOP}) lattice strain mapping obtained from geometric phase analysis (GPA) of HAADF-STEM image **a**. **c,** Line profile of lattice strain, ε_{IP} and ε_{OOP}, on the RuO_2 films extracted from GPA strain mapping **b** (blue box). These lattice strains were calculated based on the lattice parameter of reference region in TiO_2 NM (yellow box in **a**). The interface between RuO_2/TiO_2 NM is identified by the orange dotted lines in **a, b,** and **c**. In-plane lattice parameters almost invariable across the RuO_2/TiO_2 NM, indicating that the RuO_2 thin films were completely constrained from the TiO_2 NM-template. On the other hands, out-of-plane lattice parameters of RuO_2 films are about 4.5% larger than that of TiO_2 NM, and these are also uniform over the entire area of the films. It is slightly different with bulk state, where the gap of out-of-plane lattice parameter between RuO_2 and TiO_2 is about 5.4%, because the RuO_2 films were tensile-strained along the in-plane direction.

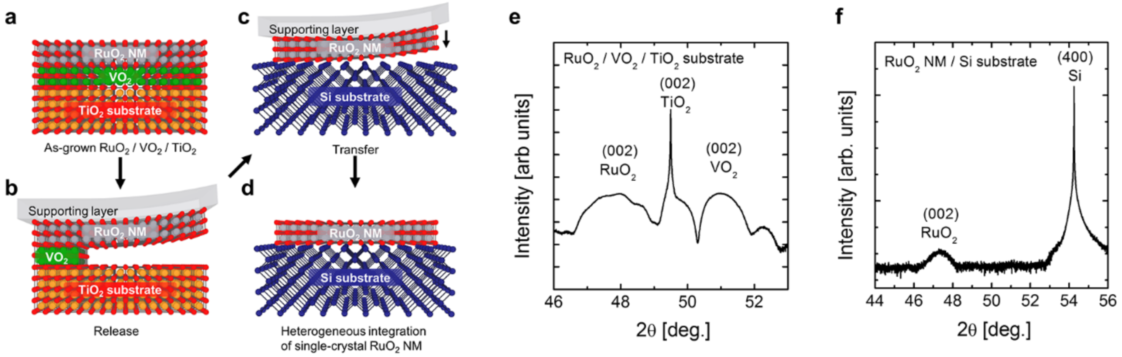

Supplementary Figure 20 | a, Schematic of an epitaxial RuO_2/VO_2 heterostructure on TiO_2 mother substrate. **b,** The VO_2 layer is dissolved in H_2O_2 to release the top RuO_2 film with the mechanical supporting layer (e.g., PDMS and TRT). **c,** The freestanding RuO_2 NM is transferred onto the Si substrate. **d,** By removing the rigid supporting layer, single-crystalline RuO_2 NM is heterogeneously integrated into a silicon substrate. Corresponded symmetric 2θ-ω XRD scans of the RuO_2/VO_2 heterostructure (**e**) and single-crystalline RuO_2 NM on Si (**f**); Symmetric 2θ-ω scan showed that (002) RuO_2 peak appeared with (400) Si substrate (**Fig. S22 f**), which confirms freestanding epitaxial RuO_2 NM was successfully transferred on the Si substrate.
